# LeGenD: High-throughput *N*-glycan profiling using explainable AI and lectin profiling

**DOI:** 10.1016/j.jbc.2026.113234

**Published:** 2026-06-04

**Authors:** Haining Li, Angelo G. Peralta, Sanne Schoffelen, Anders Holmgaard Hansen, Johnny Arnsdorf, Song-Min Schinn, Jonathan Skidmore, Biswa Choudhury, Frances Rocamora, Mousumi Paulchakrabarti, Bjorn G. Voldborg, Austin W.T. Chiang, Nathan E. Lewis

**Affiliations:** 1 Department of Bioengineering, University of California, San Diego, La Jolla, California, USA; 2 Department of Pediatrics, University of California, San Diego, La Jolla, California, USA; 3 National Biologics Facility, Department of Biotechnology and Biomedicine, Technical University of Denmark, Lyngby, Denmark; 4 Department of Microbiology and Molecular Biology, Brigham Young University, Provo, Utah, USA; 5 Glycobiology Research and Training Center, University of California, San Diego, La Jolla, California, USA; 6 Immunology Center of Georgia, Augusta University, Augusta, Georgia, USA; 7 Center for Molecular Medicine, Complex Carbohydrate Research Center, and Department of Biochemistry and Molecular Biology, University of Georgia, Athens, Georgia, USA

**Keywords:** AI, biotechnology, glycomics, glycobiology, machine learning

## Abstract

Glycosylation affects many vital functions in organisms. Thus, their measurement is critical from basic science to biotechnology, including biopharmaceutical development and clinical diagnostics. However, the throughput and cost of conventional glycan analysis can be challenging. Lectins offer an alternative approach for analyzing glycans, but they only provide glycan epitopes and not full glycan structure information. To overcome these limitations, we developed *Le*ctin to *G*lycoprofile *EN*hanced with *D*ata-driven (LeGenD), a lectin and AI-based approach, to predict dominant N-glycan structures and determine their relative abundance on purified proteins based on lectin-binding patterns. We trained the LeGenD model on 309 glycoprofiles from 10 recombinant proteins, produced in 30 glycoengineered CHO cell lines. Independent test data showed that the dominant glycosylation patterns in a given protein can be effectively determined. Further analysis using SHapley Additive exPlanations helped to identify critical lectins for glycoprofile predictions. Thus, our LeGenD approach presents an alternative platform for analyzing protein glycosylation and could complement the existing toolkits used to study glycosylation.

Glycosylation affects protein structure, function, and interactions by ensuring protein stability, proper folding, and solubility. This post-translational modification further influences protein function in diverse processes including pathogen binding, cell adhesion, signal transduction, and molecular trafficking (1, 2). Thus, changes in glycan structure can influence many biological and physiological processes (3, 4). For example, changes in glycosylation can modulate inflammatory responses, facilitate viral immune escape, aid metastasis, orchestrate apoptosis, and participate in the pathophysiology of various genetic and infectious diseases (5, 6). Comprehensive profiling of protein glycosylation is critical for biomedical research, including the study of how single sugar residues can considerably alter protein function and activity (7, 8, 9), and the identification of prognostic and diagnostic biomarkers for diverse diseases and genetic defects (10, 11, 12).

Several methods can be used to measure glycan features, such as HPLC (13), capillary electrophoresis (14, 15), MS (16, 17), nuclear magnetic resonance (18), lectin arrays (19, 20), and enzyme-linked immunosorbent assays (21). While each continues to provide invaluable information addressing specific biological questions, glycomics continues to trail behind the strides achieved by other omics, especially in next-generation sequencing of nucleic acids (22, 23, 24, 25, 26). Considering the growing needs for glycobiology research, alternative techniques for assaying and quantifying glycan structures could be beneficial.

Lectins are carbohydrate-binding proteins that recognize mono- and oligosaccharide structures and can be applied in diverse analytical formats (27, 28, 29). to measure glycan features (19, 30). Despite their value for quantifying glycosylation patterns across diverse samples and conditions, lectin arrays and assays remain underutilized, and have yet to be used to measure entire glycan structures (19). Moreover, samples with considerably different glycan compositions can produce similar lectin profiles (LPs), making these differences difficult to observe (31, 32). Lastly, although successful work has elucidated many lectin-binding motifs, lectin binding affinities can remain ambiguous if they have only been tested on single glycan structures or on a single type of array, nor tested on variable external factors (33, 34, 35). Therefore, it is challenging to leverage lectin-based approaches to comprehensively elucidate intact glycoform structures in a given glycoprotein, and there is a clear need for more data-driven innovations.

In recent years, artificial intelligence (AI) models have been applied to many biological challenges to analyze diverse data (36), and complex omics data for diverse applications, such as the reconstruction of metabolic pathways (37), drug discovery, and biomarkers (38, 39). Glycomics undoubtedly involve the deciphering of hidden connections, especially given the ambiguity found in glycan structures (from their branching nature and inclusion of repeating structural units) and the various molecules that bind to glycans. Several studies have applied AI tools (40, 41, 42). to glycomics research. Essentially, these studies successfully extrapolated glycan-binding properties without performing costly and labor-intensive experiments by harnessing neural-network models. Additionally, AI-based approaches (43, 44). have been adopted to illustrate the structure and functional relationships of glycans. To facilitate the interpretation of such models applied in glycobiology, explainable AI could be used with the black-box AI models. With such explainable AI methods, explainability is generally defined as the assignment of meaning between model input and output using model-agnostic methods. One such method, Shapley Additive Explanations (SHAP) (45, 46, 47). is an approach, based on game theory that dissects the individual predictions of AI models, allowing the computation of Shapley values for the sets of essential input features that guide local and global model result explainability. For black-box AI models in general, explainability as a concept is essential for both i) understanding the real-world significance of the relationship observed between input features and model results, and ii) guiding the refinement of said models to improve upon the predictive accuracy for the objective of interest. As such, the explainability of AI-based methods in glycobiology is necessary to both resolve mechanisms of glycan synthesis and evaluate glycomic data.

Here, we present a method called *Le*ctin to *G*lycoprofile *EN*hanced with *D*ata-driven methods (LeGenD), which uses an artificial neural network (ANN) model to quantify major *N*-glycan structures using enzyme-linked lectin assays (ELLA). Specifically, we generated a large dataset of diverse ultra-performance liquid chromatography (UPLC) glycan profiling experiments using a genetically engineered CHO cell panel. By harnessing established lectin specificities (48), we simulated the lectin-binding profiles of the 309 collected glycoprofiles (GP). We fine-tuned the data using paired LPs and glycan profiles of additional proteins, and used this to train the LeGenD model. The model was then validated using independent lectin profiling data from recombinant human alpha1-antitrypsin (rhA1AT) and plasma-derived alpha1-antitrypsin (pdA1AT). GPs predicted by ANN were generally consistent with those derived from UPLC, although there were minor differences in the accuracy of certain glycan species. Finally, we used SHAP to identify the dominant lectins responsible for reconstructing the GP. Taken together, LeGenD opens the door to an era of AI-enabled restoration of glycan structures through high-throughput and cost-effective lectin analysis, thereby demonstrating the untapped potential of lectins for glycan analysis.

## Results

### Lectin profiling cannot unambiguously predict glycoprofiles

**Figure 1:**
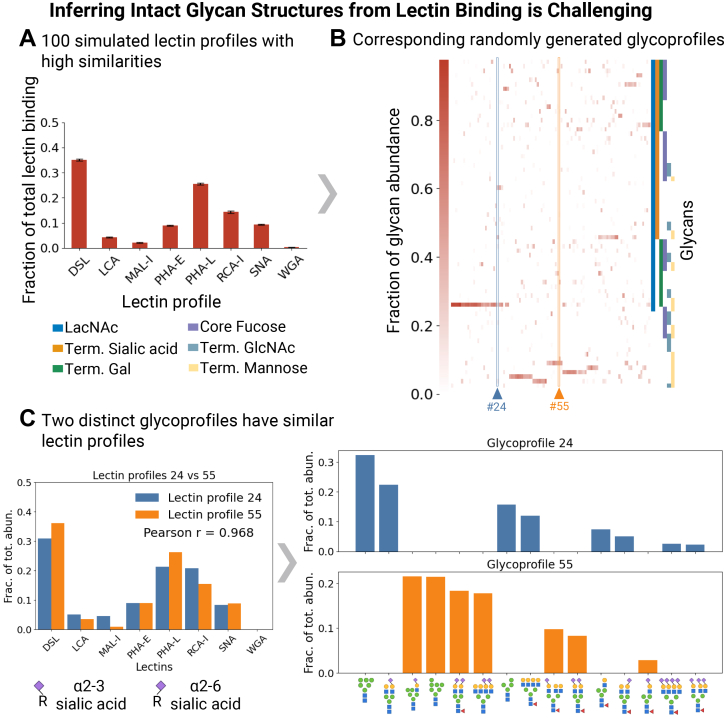
**Predicting GPs from LP is under-constrained.** From 4722 randomly generated GPs, we simulated their LPs and then plotted the GPs corresponding to the 100 most similar simulated LPs (Pearson correlation > 0.95). *A*, bar plot of mean LP representing their fraction of total lectin binding, with standard error bars showing the variability for each lectin. *B*, the heatmap shows the similar candidate GPs (*black squares*) derived from LPs after clustering using the Voorhees algorithm. The glycan features are annotated on the *right*. *C*, two representative LPs from panel A plotted together alongside their corresponding GPs, while their lectin similarity is high (Pearson r = 0.968), their GP is distinct (cosine distance = 1). All data are simulated. GP, *g*lycoprofile; LP, lectin profile.

Lectins are valuable reagents for measuring glycan features; however, the diversity of glycan features that can be oriented in glycan structures (29, 49, 50). has limited the ability to use lectins to unambiguously determine glycan structures. Furthermore, many lectins have secondary specificities, thereby increasing the potential glycan structures that they bind to (51). To estimate the scale of this problem, we aimed to quantify the diversity of GPs that could be obtained from a single lectin binding pattern. We started to generate random GPs from a set of 74 known glycan structures that were measured from a panel of 10 glycoproteins expressed in 30 glycoengineered Chinese hamster ovary (geCHO) cell lines (see Methods). For each sampled GP, we simulated its lectin-binding profile using established lectin binding rules (see Methods) and retained those whose simulated profiles were highly similar to the first one generated (Pearson r > 0.95). A total of 4722 GPs were sampled to obtain 100 that satisfied this criterion. Figure 1, *A* and *B* depicts these 100 examples with both their LPs and corresponding GPs, showing that compositionally distinct GPs can yield nearly identical lectin signatures. For instance, GPs #24 and #55 exhibit nearly indistinguishable lectin-binding patterns (Pearson r = 0.968, Fig. 1*C*) yet correspond to compositionally distinct structures (cosine distance = 1).

To characterize diversity among the retained GPs, we performed hierarchical clustering using the Voorhees algorithm, which revealed at least seven coherent clusters, each marked by a distinct dominant glycan. However, each cell constrains the GPs it produces based on species, enzyme repertoires, monosaccharide availability, and protein structure; thus the range of possible GPs is greatly reduced (52, 53). Because the quantitative influence of these constraints is not well understood, we hypothesized that training an ANN on highly diverse GPs would capture these inherent biosynthetic constraints and enable accurate GP prediction from lectin data.

### Simulated and experimental lectin-binding profiles are similar

**Figure 2:**
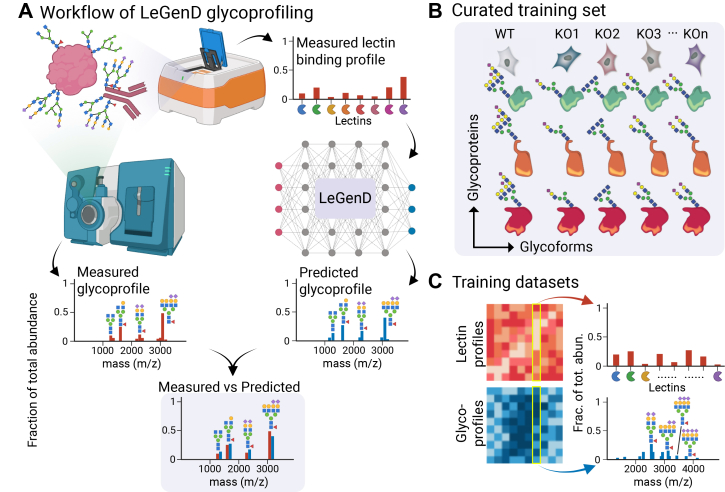
**Predicting relative N-glycan abundance using machine learning.***A*, workflow of LeGenD utilizing neural networks to predict N-GPs based on lectin-binding profiles. *B*, 309 N-GPs were obtained from 10 glycoproteins expressed in a large panel of 30 glycoengineered Chinese hamster ovary cells, yielding a comprehensive view of N-glycans for training LeGenD. *C*, matrix representations of training datasets consisting of LPs and GPs. GP, *g*lycoprofile; LeGenD, *Le*ctin to *G*lycoprofile *EN*hanced with *D*ata-driven.

**Figure 3:**
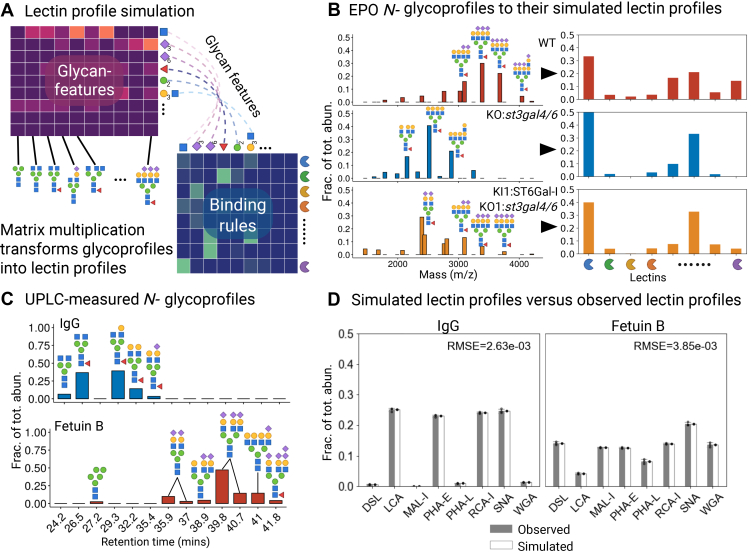
**Simulating LPs for model training data.***A*, schematic approach for simulating LPs from GPs through matrix multiplication using lectin-binding rules. Glycans are mapped to lectins by decomposing each glycan into its constituent glycan features recognized by each lectin. *B*, simulation of GPs with their respective LPs. *C*, UPLC-derived N-GPs for IgG from human sera and bovine Fetuin B, Validation of simulated lectin profiling by comparing simulated LPs of IgG and Fetuin B, generated from their UPLC-derived N-GPs. *D*, validation of lectin enzyme-linked lectin assay data for Bovine Fetuin B samples and IgG for human sera, generated from UPLC analysis, against actual enzyme-linked lectin assay experiments with standard *error bars*. The comparison revealed low root mean square error values, confirming the accuracy of our simulation. GP, *g*lycoprofile; LP, lectin profile; UPLC, ultra-performance liquid chromatography.

LeGenD uses an ANN to connect patterns of lectin binding to GPs (Fig. 2*A*). However, there is a paucity of available datasets containing detailed lectin-binding patterns and their corresponding glycan structural information. To address this challenge, we used 30 geCHO cell lines comprising different combinations of gene knockouts and knockins of 17 glycosyltransferases (Fig. 2*B*). These cell lines were used to produce a range of different glycoforms of 10 recombinant glycoproteins, including monoclonal antibodies, fusion proteins, cytokines, and enzymes (54). We performed N-glycan analysis using HILIC-UPLC profiling, which yielded 309 GPs (Supplementary Data 1). We then simulated the LPs from this large GP dataset (Fig. 3) to form our training dataset (Fig. 2*C*).

The simulation of the lectin binding profiles was performed by multiplying a glycan-feature matrix (where each glycan is decomposed into counts of expressed features with each glycan feature annotated as per Linearcode (55)) by a binding-rules matrix (Fig. 3*A*) (Supplementary Data 3). Figure 3*B* demonstrates examples of simulated LPs from measured EPO GPs. We then aligned some simulated LPs with their experimentally observed counterparts to enhance the credibility of our simulated LPs.

While our simulated lectin-binding patterns are based on published binding factors (48), it is imperative to test whether the values are consistent with our experimental data. This is especially crucial to validate our assumption that the binding intensity is proportional to the amount of glycan features to which the lectin binds. Thus, we validated this using IgG from human sera and bovine Fetuin B as representative glycoproteins, which contain different *N*-glycan structures((56, 57, 58)). The *N*-glycans of both glycoproteins were characterized separately using UPLC (59, 60), and the relative amounts of respective *N*-glycans were estimated from the peak regions of the fluorescence chromatograms (Fig. 3*C*). Adhering to our simulation rules, simulated LPs were constructed for each glycoprotein. Concurrently, we used ELLA to quantify N-linked glycan characteristics by selecting lectins with glycan epitopes consistently present in both glycoproteins, serving as a benchmark for analytical development (see Methods for details) (Table S1). The selected lectins were also evaluated for cross-reactivity with O-linked glycans by analyzing lectin specificities from the CFG (https://www.functionalglycomics.org) microarray data (Fig. S1) and O-glycan modifications in Fetuin B using MALDI-TOF (Fig. S2). Although the lectin microarray suggested potential cross-reactivities for certain lectins, O-glycan profiling of Fetuin B revealed the absence of their primary binding epitopes. Thereafter, experimentally-derived LPs were generated (Supplementary Data 2) (Fig. 3*D*). The comparison indicated strong correspondence between the simulated and experimental LPs. Moreover, linear regression analysis allowed us to align the simulation data with the observed data for increased accuracy. Following the application of a linear regression model, our results demonstrate a significant resemblance of simulated LPs derived from fluorescence chromatograms data to the actual LPs for both IgG root mean square error (RMSE = 2.63e-03) and Fetuin B (RMSE = 3.85e-03) (Fig. 3*D*). Hence, the simulated LPs were expected to be suitable training data for LeGenD in subsequent experiments.

### LeGenD accurately predicts *N*-glycosylation in A1AT

**Figure 4:**
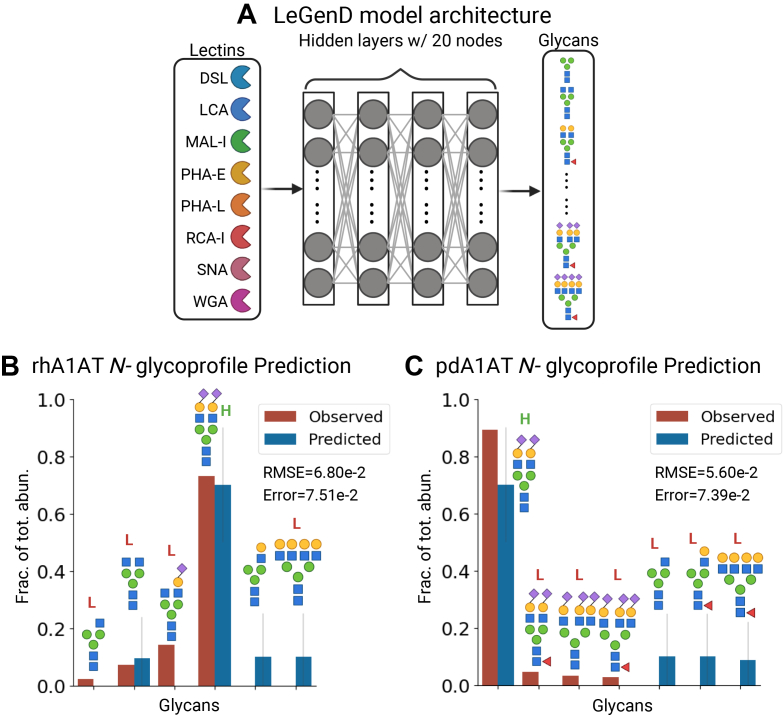
**Predicting the A1AT N-GPs from measured lectin binding profiles.***A*, the architecture of the LeGenD neural network model, consisted of four fully connected hidden layers with 20 nodes each. *B*, comparison between the experimentally observed recombinant human alpha1-antitrypsin-GP and the LeGenD-predicted N-GP. The *red bar* represents the GP obtained using UPLC, whereas the *blue bar* represents the predicted GPs obtained by LeGenD using observed lectin assays as inputs. The fraction of total abundance for each glycan structure was determined. *Error bars* indicate standard error. The symbol for each glycan follows the SNFG standard. H/M/L labels indicate high-, medium-, and low-confidence predictions, respectively. *C*, comparison between the experimentally observed plasma-derived alpha1-antitrypsin-GP and the LeGenD-predicted N-GP. GP, *g*lycoprofile; LeGenD, *Le*ctin to *G*lycoprofile *EN*hanced with *D*ata-driven; UPLC, ultra-performance liquid chromatography.

We built a fully connected ANN model (Fig. 4*A*) trained on 309 experimentally measured N-GPs mentioned previously. The resulting dataset encompassed 74 annotated N-glycan structures—the complete set of experimentally derived glycans observed across our training data. Since our ANN is a supervised model, it can only predict glycans observed during training; novel glycans not represented in the dataset will be approximated as the closest matching known structure. This reflects a current limitation of our framework; however, the 74 structures encompass the dominant N-glycans we have measured in CHO cells and are well-supported by our comprehensive collection of glycosyltransferase knockouts and knockins. Each GP was paired with its corresponding simulated lectin-binding profile, forming the input–output pairs used for ANN training. To optimize model performance, we performed hyperparameter tuning and confirmed a model architecture consisting of four hidden layers and 20 nodes in each layer performed the best (RMSE of −3.80E-02) (see Methods for full detail). The detailed hyperparameter sets tested are listed in Table S2.

This model was evaluated for its ability to computationally predict N-glycans from the ELLA-derived LPs. We first provided rhA1AT and pdA1AT LPs to the ANN to predict the identity and abundance of the rhA1AT and pdA1AT N-glycans (61) (Fig. 4, *B* and *C*). To denoise, we employed a threshold fractional abundance of 0.02, and then normalized the predictions to have a summed abundance of 1. The predictive accuracy of the GPs was evaluated by comparing observed and predicted values, as illustrated in Figure 4. For the rhA1AT GP, the model achieved RMSE of 0.068 and an error of 0.075, indicating moderate agreement between predictions and experimental data. In contrast, the pdA1AT GP demonstrated markedly higher precision, with an RMSE of 0.056 and an error of 0.0739, reflecting strong alignment between predicted and observed trends.

To aid interpretation of individual glycan predictions, we further annotated each predicted glycan with model-intrinsic confidence. Confidence labels were computed directly from the model-predicted abundance distribution using fixed logit-separation and abundance thresholds, without comparison to the experimentally observed GP. Predicted glycans were labeled as high-confidence (H), medium-confidence (M), or low-confidence (L), allowing users to distinguish dominant, well-supported predictions from lower-confidence predictions that should be interpreted more cautiously. Our results suggest that the model performs most reliably for more dominant glycoforms. Overall, the findings underscore the model's capability to capture key features of GPs while identifying areas for refinement.

### Crucial lectins can be identified using SHAP

**Figure 5:**
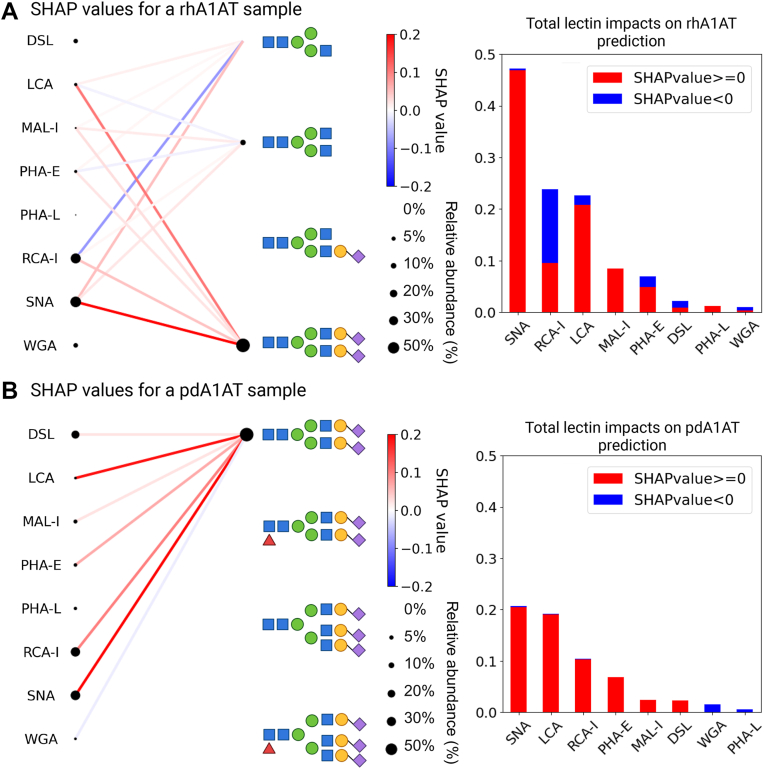
**The SHAP values explain the contribution to prediction from each lectin.** In the bipartite network on the *left*, SHapley Additive exPlanations values of lectins and their glycan structures in (*A*) recombinant human alpha1-antitrypsin and (*B*) plasma-derived alpha1-antitrypsin are displayed. The color scale represents SHAP values, whereas the *dot size* indicates the relative abundance of a lectin or glycan. To the *right*, a bar graph breaks down the sum of the SHAP values, illustrating the contribution of each of the eight lectins. *Purple* diamonds oriented towards the *right* and *left* represent ɑ-2,6-linked sialic acids, respectively. SHAP, Shapley Additive Explanation.

To interpret the “black box” model in LeGenD, we employed a model-agnostic SHAP analysis (45, 46, 47). This approach identified key lectins and their associated glycan epitopes contributing to GP predictions derived from ELLA lectin-binding profiles. For rhA1AT (Fig. 5*A*), SHAP analysis highlighted SNA, RCA-I, and LCA as the most influential lectins, with absolute SHAP importance values of ∼0.5 and ∼0.25 above the background (averaged values of glycan abundance in training dataset), respectively. In pdA1AT (Fig. 5*B*), SNA and LCA retained prominence, while PHA-L and WGA were among the least predictive lectins in both proteins.

SNA’s positive SHAP values align with its specificity for α-2,6-linked sialic acid (62, 63), a well-documented modification on A1AT (61, 64). This reinforces the model’s ability to link SNA’s binding profile to sialylation levels. LCA, which binds fucosylated glycans, also displayed positive SHAP values. Here, diminished LCA signals—reflecting reduced fucosylation—correlated directly with the model’s prediction of abundant afucosylated glycans. This relationship underscores the model’s capacity to interpret lectin-binding patterns in a biologically meaningful way, where low LCA activity serves as a predictor of afucosylation.

MAL-I, targeting Siaα2-3Galβ1-4GlcNAc epitopes (48, 65, 66), contributed positively despite the absence of its canonical glycan in A1AT. The model likely associates MAL-I’s binding behavior with structural features co-occurring in the dataset, enabling accurate predictions even in the absence of its primary epitope. These results highlight the framework’s ability to integrate diverse lectin signals, translating them into coherent glycomic insights without relying on indirect interpretations.

By anchoring SHAP values in A1AT-specific glycan biology, the analysis demonstrates how lectin-binding profiles inform predictions. For example, the bipartite plot for pdA1AT LCA (Fig. 5*B*) illustrates that low LCA signals (indicative of afucosylation) strongly align with high predicted afucosylated glycan levels—a direct reflection of established biochemical principles. This approach ensures clarity and relevance, emphasizing the model’s fidelity to empirical data.

## Discussion

Here, we introduced LeGenD, a method for deciphering lectin-binding patterns and predicting the identities and quantities of the dominant N-glycans in a given sample. Our AI model, trained with diverse glycoproteins, captures inherent relationships between glycans as determined by the cell. It provides sufficient resolution and sensitivity for quantifying highly-expressed glycans and is compatible with different analytical platforms, enabling miniaturization and high-throughput glycan characterization. LeGenD can complement current analytical methods that can encounter challenges in deciphering glycan structural features due to isobaric monosaccharides, stereochemistry (67, 68), and the need for comprehensive preliminary structural assignments and subsequent validation using orthogonal technologies implemented by experts in glycoanalytics (30, 69). Although several studies have attempted to profile glycans holistically (70) or to predict single glycans from lectin binding patterns (35), mapping these patterns to an actual GP is a highly under-constrained problem; wherein nigh infinite GPs could have the same LP (Fig. 1). However, LeGenD overcomes this challenge by using AI with a large glycomics training set to successfully compute an N-glycan profile based on a lectin-binding profile. In conjunction with explanatory AI approaches, such as SHAP analysis, we also identified the most predictive lectins.

In the present study, we demonstrated that ANNs can be used to identify an accurate GP from a lectin-binding profile, despite the immense diversity of glycans that could exist based solely on known enzyme functions. Glycans are highly complex, highly branched, nonlinear biopolymers that are synthesized through seemingly stochastic interactions between glycosyl hydrolases and glycosyltransferases (71, 72, 73). However, it is anticipated that the physicochemical properties of the cell and the glycosylated proteins will limit the range of possible glycan structures and possibly which ones are more likely to co-exist in any given sample (6, 74, 75). Our study shows that ANNs capture these constraints on glycan synthesis, thus enabling the accurate determination of the correct GPs despite the expected theoretical complexity. While LeGenD currently performs most reliably for high-abundance glycans, this focus aligns with the biologically and analytically dominant components of recombinant glycoproteins. Accurate quantitation of these major glycoforms is critical for assessing product quality and structural integrity, and thus remains a meaningful benchmark for predictive performance. Lower-abundance glycans may require expanded lectin panels or data augmentation strategies to achieve comparable resolution in future iterations. To make this limitation transparent to users, we now provide confidence tiers for individual glycan predictions. These annotations are intended to flag predictions that are strongly supported by the model *versus* those that should be treated as tentative and more rigorously validated by orthogonal glycoanalytical methods before making conclusions dependent on low-confidence glycans.

While conventional neural networks have been successful here and in other studies for studying glycosylation patterns and processes (76, 77, 78, 79), further developments in A.I. have presented new model topologies that could further refine technologies, such as ours, for more accessible technologies in glycobiology. For example, glycans are well-suited for graph neural network (GNN) applications (42, 43, 80, 81). Indeed, GNNs leverage the full molecular structure or even the geometry for inputs, and the GNN itself learns informative molecular representations to predict the given target properties (82, 83, 84). In the context of glycans, they can comprehensively account for monosaccharides and their linkages, represented as nodes and edges, respectively, to form “glycowords” (81, 85). This strategic representation facilitates the capture of unique structural features and contextual information pertaining to glycans including branching substructures, anomericity, and various types of linkages (85). GNNs have been successfully employed for motif exploration within glycan substructures, enabling categorization by *O*-/*N*-linkage, immunogenic properties, evolutionary origin, and viral protein recognition (43, 81). In this work, however, our focus was on establishing a proof-of-concept framework demonstrating the feasibility of predicting GPs from lectin data, rather than optimizing predictive performance or benchmarking across architectures. Therefore, we anticipate that integrating alternative model architectures beyond standard neural networks will further enhance applications for studying glycan structural patterns. These results are highly compatible with our workflow, resulting in an improved prediction performance.

SHAP analysis provided further insights into glycan epitopes that require better reagents for quantification. For example, we found that WGA and PHA-L lectins, which target complex epitopes such as β-1,4-GlcNAc-linked and sialic acid-containing residues (86), tri-, and tetraantennary structures (48), did not significantly improve the predictive performance of LeGenD for rhA1AT and pdA1AT. We hypothesized that, while these lectins can recognize various structures, such as internal complex *N*-glycan structures, there is a need for more specific carbohydrate-binding proteins to enhance the model's overall performance. Therefore, future studies are needed to identify and screen proteins with better binding specificities to the internal structure of glycans. Another important goal for development is to increase the available repertoires of lectins that can be utilized. Not all lectins perform optimally during *in vitro* analyses (28, 48, 87). However, recent advances have largely demonstrated alternatives to overcome this limitation (74, 88, 89). Therefore, we anticipate the development of new recognition molecules will aid in accelerating the emergence of high-throughput glycomics.

While SHAP effectively reveals the individual contribution of each lectin to GP prediction, its primary utility is that of an audit of model behavior. This audit facilitates the biological interpretation of model prediction in relation to supplied features. Specifically, when visualized as bipartite plots (Fig. 5, *A* and *B*), the local SHAP scores (Shapley values) provide accessible explanations of how lectin abundances influence the model's predictions for rhA1AT and pdA1AT GPs independently. These explanations reflect the average contribution of a feature value to the prediction across different coalitions, rather than indicating how the prediction would change if the feature were removed from the model. Consequently, SHAP provides a quantitative rationale for the model's prediction based on the supplied features, similar to explainable models such as linear regression models (47). Therefore, the results should be interpreted as correlations rather than causal relationships (90). In summary, SHAP enhances our understanding of the data beyond just the weights from a neural network. Lastly, SHAP will also be valuable in future studies for evaluating different models and improving their explainability, ultimately leading to more accurate predictions and insights from LeGenD.

In addressing the limits of traditional methods for assessing glycan structures and abundances, such as throughput constraints and sensitivity concerns (91, 92), particular examples such as microtiter plate-based techniques (ELLA/ELISA) highlight the need for improvement. These methods, which detect only one target within a single reaction well (93, 94). and struggle to achieve high sensitivity comparable to PCR due to their inability to utilize direct amplification techniques (95), underscore the importance of finding solutions to enhance the development of *in vitro* glycan research tools. In response, the integration of glycan binding proteins like lectins with DNA barcoding is promising. This approach enables the identification of lectin-glycan interactions *via* PCR or Next Generation Sequencing, facilitating highly-sensitive quantitative glycan analysis (96, 97). Furthermore, within the expanding omics paradigm, multiplexing methodologies are becoming increasingly vital for evaluating glycan complexity and diversity at high throughput scales.

LeGenD is poised to emerge as a rapid, streamlined technology for discerning highly abundant glycoforms across biotherapeutic batches, enabling high-throughput glycan mapping and monitoring. By combining the quantitative precision of structural composition analysis (*e.g.*, chromatography and MS) with the linkage-specific insights of lectin arrays, our method addresses the unmet need for platforms that track predominant glycosylation changes across diverse contexts, offering potential to establish clinically relevant N-glycan signatures. In practical use, the confidence annotations can guide users toward dominant, high-confidence glycan calls while distinguishing lower-confidence minor species that may require confirmatory analysis. While orthogonal separation-based methods can validate LeGenD’s predictions for critical applications, the framework’s versatility allows extension to other glycosylation types (*e.g.*, O-glycans) and formats such as lectin arrays (98, 99, 100), microfluidics (101), or Next Generation Sequencing (97, 102, 103), each requiring tailored training datasets. To broaden its utility, future iterations would benefit from expanded training data generated *via* multivariate probability distributions (*e.g.*, Dirichlet distributions), encompassing understudied features like bisecting GlcNAc, non-core fucose, modified sialic acids, and polysialic acids. Though focused primarily on dominant N-glycoforms, LeGenD demonstrates immediate practical value by accurately identifying major glycans essential for biotherapeutic characterization. This capability can accelerate clone triaging, media optimization, and glycoform quality assessment in biomanufacturing. With expanded training data and refined lectin panels, the framework is positioned to improve sensitivity for lower-abundance structures while maintaining its core strength in predicting the glycans most critical to product quality and efficacy.

In summary, as awareness of the importance of glycosylation increases, new technologies are needed to increase the accessibility and throughput of glycan research tools. LeGenD provides a complementary concept that with further development could aid in the proliferation of glycoanalytics in routine biological research. Indeed, leveraging AI in glycoanalytics can help manage the complexity of this field, thereby enabling a much larger user base for glycomics and allowing researchers to link changes in glycosylation with phenotypic changes.

## Experimental procedures

### Lectin selection

We selected lectins capable of providing a wide coverage across a broad spectrum of N-glycoform epitopes found in our glycoprofiling training dataset. 13 preliminary lectins were chosen (data not shown). However, by removing weak binding lectins or are unavailable in biotinylated form, preventing the use of multimerization to increase *in vitro* binding strength, as well as the inability to perform linear regression during LP simulation resulted in refining our selection to an array eight lectins that still enables a comprehensive characterization of N-glycosylation features (48, 104). Specifically, these lectins can distinguish glycan structures such as branches with up to four branches (GlcNAc-β1,2/4/6), LacNAc elongation (GlcNAc-β1,3), epitope monosaccharides (*e.g.*, fucose), and high mannose structures (see Table S1).

### *N*-glycan release and preparation from glycoproteins for LC-based glycan characterization

150 μg IgG (from human serum; ≥ 95% SDS-PAGE, Sigma-Aldrich, St Louis, MO) and 300 μg fetal bovine Fetuin B (purity ≥90%, Sigma-Aldrich) were each treated with a mixture of PNGase F (purity ≥95%, based on SDS-PAGE and ESI-MS, NEB) following the manufacturer’s protocol.

### IgG and fetuin B *N*-glycan purification and procainamide labeling

The *N*-glycans obtained by PNGase F treatment of Fetuin B and IgG were purified using C18 Sep-Pak 1 cc (Waters) columns. To eliminate salts and hydrophilic species, the purified samples were subjected to an additional cleanup step using graphite cartridges. The C18 Sep-Pak column was initially conditioned with 1 ml of 10% aq MeOH + 1% HOAc three times, followed by sequential addition of 50% aq MeOH, 100% MeOH, and Chloroform. Subsequently, the column was washed twice with 1 ml of 100% MeOH, 50% aq. MeOH, and 1 ml of H_2_O five times. Similarly, HyperSep Hypercarb 25 mg Porous graphitic carbon columns (Thermo Fisher Scientific) were conditioned with 1 ml of 1% trifluoroacetic acid, 50% aq acetonitrile (ACN), and 100% ACN three times, followed by 1 ml 50% aq ACN twice, and with 1 ml H_2_O three times. The Sep-Pak and Porous graphitic carbon columns were further washed with 1 and 3 ml of H_2_O, respectively. Finally, *N*-glycans were eluted using 3 ml of 30% aqueous ACN containing 0.1% trifluoroacetic acid, and the elution fraction was collected and lyophilized for further analysis.

The reconstituted *N*-glycans were labeled with procainamide *via* reductive amination, as described by Xie *et al.* (105). Briefly, a procainamide solution was prepared by dissolving procainamide in sodium cyanoborohydride in a DMSO:acetic acid:H_2_O (280:120:100 v/v/v) solution and mixed thoroughly. Labeling solution was added to each sample and incubated at 65 °C for 1 h. ACN was mixed with each sample, and SPE cleanup was performed using GlycoClean S-Cartridges (ProZyme). After cleanup, the samples were washed thrice with ACN. Finally, the procainamide-labeled *N*-glycans were eluted with H_2_O.

### UPLC analysis of *N*-glycans from IgG from human sera and fetal bovine fetuin B

*N*-glycan analysis was performed using a Waters Acquity UPLC system attached to a fluorescence detector (Besson Scientific). Specified quantities of *N*-glycan were tagged with the fluorophore procainamide and profiled on a Glycan BEH amide column (150 mm × 2.1 mm × 1.7 um) (Waters). A gradient mixture of solvents containing A:100 mM ammonium formate (pH-4.5) and B: ACN was used to profile glycans. The solvent gradient conditions used are listed in Table 1. Detection was performed using a fluorescent detector set at excitation and emission wavelengths of 310 and 370 nm, respectively. The column temperature was set at 60 °C and the autosampler temperature was maintained at 10 °C. The data were processed using Chromeleon software (Thermo Fisher Scientific; https://www.thermofisher.com/us/en/home/industrial/chromatography/chromatography-data-systems-cds.html).

**Table 1:** Ultra-performance liquid chromatography solvent mixtures for IgG and Fetuin B profiling

Time (min)	Flow (ml/min)	%A	%B
0	0.5	22	78
10	0.5	22	78
52	0.5	47	53
55	0.25	100	0
57	0.25	100	0
60	0.5	22	78
70	0.5	22	78

### CHO-derived recombinant protein purification and HILIC-UPLC *N*-glycan analysis

Herceptin, Rituximab and Enbrel were purified by protein affinity chromatography. For each protein glycoform, a 1-mL MAbSelect Extra column (Cytiva) was equilibrated with 5 column volumes of 20 mM sodium phosphate, 0.15 M NaCl, pH 7.2. Next, 30-mL supernatant was loaded, the column was washed with 20 column volume of 20 mM sodium phosphate, 0.15 M NaCl, pH 7.2, and the protein was eluted using 0.1 M citrate, pH 3.0. The elution fractions (0.5 ml) were collected in deep-well plates containing 100 μl of 1 M Tris at pH 9 per well.

Alpha-1-antitrypsin, butyrylcholinesterase, protein Z, serpin A5, serpin A10, serpin C1, and EPO, all C-terminally tagged with the HPC4 tag (amino acids EDQVDPRLIDGK), were purified over a 1-mL column of anti-protein C affinity matrix according to the manufacturer’s protocol (Roche, cat. no. 11815024001). 1 mM CaCl2 was added to the supernatant, equilibration buffer, and wash buffer, respectively. The proteins were eluted in 0.5 ml fractions using 5 mM EDTA in the elution buffer.

For all proteins, elution fractions containing the highest protein concentration were concentrated using Amicon Ultra centrifugal filter units (MWCO 10 kDa). 12 μl of concentrated protein solutions (concentrations varying between 0.1 and 1 mg/ml) were subjected to *N*-glycan labeling using the GlycoWorks (https://www.waters.com/nextgen/us/en/products/application-kits/glycoworks-kits.html?xcid=ppc-ppc_25589&gad_source=1&gad_campaignid=14746094371&gbraid=0AAAAAD_uR00Ug9a4Kjc7Dn4FxtFNLqts1&gclid=CjwKCAjw0dPRBhAPEiwAE5vTTqHAC7S4UsaIOXa1R8tsHAVn2kn-XKA4crNTwrrrjPJgk6r1ANG9tBoCeG4QAvD_BwE) RapiFluor-MS *N*-Glycan Kit (Waters). Labeled glycans were analyzed by HILIC-FLR using an ACQUITY UPLC Glycan BEH Amide column (2.1 × 150 mm, 1.7 μm) (Waters) mounted on an Ultimate 3000 UPLC system and a Fusion Orbitrap mass spectrometer (Thermo Fisher Scientific). ACN (100%) and ammonium formate (50 mM, pH 4.4) were used as mobile phases. The data were processed using Thermo Xcalibur software v4.3 (Thermo Fisher Scientific; https://www.thermofisher.com/us/en/home/industrial/mass-spectrometry/liquid-chromatography-mass-spectrometry-lc-ms/lc-ms-software/lc-ms-data-acquisition-software/xcalibur-data-acquisition-interpretation-software.html).

### Analysis of *O*-glycan analysis by MALDI-TOF mass spectrometry

O-glycan analysis was performed similarly according to Zhong *et al.* (106). Briefly, a known amount of Fetuin B was treated with 50 mM NaOH and 1 M NaBH4 for 16 h at 45 °C. The reaction mixture was slowly neutralized with ice-cold 30% acetic acid, purified using Dowex 50-X cation exchange resin, and lyophilized. Excess boric acid generated was removed by co-evaporation with acidified methanol, followed by methanol. The *O*-glycan was then purified using a C18 cartridge, methylated, and analyzed after permethylation. Mass spectral data were acquired using a Bruker AutoFlex mass spectrometer in positive reflectron mode and analyzed and annotated using GlycoWork Bench.

### Enzyme-linked lectin assay

96-well high-bind microplates (Corning) were coated overnight at 4 °C with 100 μl of 10 μg/ml sample of interest using carbonate coating buffer (100 mM NaHCO_3_, 30 mM NaCO_3_, pH 9.5). The plate was then blocked with a blocking buffer (1 × PBS with 2% w/v polyvinylpyrrolidone) for 1 h at 37 °C. Biotinylated lectins (Vector Labs) were diluted to 10 μg/ml in binding buffer solution (10 mM Hepes, 0.15 M NaCl, 0.1 mM CaCl_2_, pH 7.5) and added to the plate for 30 min at room temperature. The avidin-biotinylated HRP complex (ABC reagents; Vector Lab) was subsequently added according to the manufacturer’s instructions. Each step was washed thrice with PBST (Sigma-Aldrich). HRP activity was determined by incubation with TMB (Sigma-Aldrich) and H_2_O_2_ at room temperature for 1 to 10 min and quenching with 1 MH_3_PO_4_. The color was detected using a Biotek Synergy Mx plate reader (Agilent) at 450 nm.

### Relative and absolute quantities of *N*-glycans

The relative quantity (%) of each *N*-glycan was calculated from the sum of the individual UPLC peak areas in the chromatogram (107). Each chromatogram area was generated using LC-ESI-HCD-MS/MS, and the quantity (%) of each *N*-glycan (>0.1%) was determined relative to the total amount of *N*-glycans (100%) (107). The absolute quantity of non-overlapping glycan peaks was estimated from the fluorescence intensity of the glycan peak in the UPLC chromatogram, using a linear calibration curve (r2 = 0.99) generated from various concentrations of AB or ProA. The quantities of the remaining glycans were calculated using the relative quantities of Fetuin B and IgG from the absolute quantity represented by the glycan peak (107). The glycan quantities of rhA1AT and pdA1AT was previously reported (61).

### Analysis of UPLC chromatograms

The main glycan peaks were identified and annotated for each profile. The total abundance fraction of each *N*-glycan was calculated from the fraction of individual UPLC peak areas relative to the total area. Owing to some unannotated peaks, most samples did not reach a summed abundance of 1. Some poorly annotated samples identified as outliers by the Matplotlib boxplot (108) were excluded. We normalized the rest of the GP dataset for each sample to obtain a total abundance of 1. The raw UPLC peak images provided only the overall glycan composition. Therefore, detailed structures and glycosidic linkages were derived based on references from the literature(109, 110). Finally, we converted the glycan annotation into linear code (111) style for our choice of nomenclature.

### Analysis of ELLA binding profiles

The assay signal for each lectin was calculated as the average background-subtracted intensity for each sample. Subsequently, the data for each sample were normalized to a total abundance of 1.

### Lectin profile simulation

Based on the literature, we selected eight lectins that mutually recognize 10 distinctive common *N*-linked glycan features (Table S1). These eight lectins were selected based on two critical factors. First, the selected set of lectins should be able to recognize most of the *N*-linked glycans found in the GP dataset. Second, these lectins exhibit high specificity and affinity towards their intended glycan epitopes.

For any given GP, LP can be generated by using Equations (1), (2) below.

(1) $${Lpg}_{i,j}={GF}_{I,K}*W_{k,j}$$

*LPg*_*i,j*_ is the lectin binding profiles for given glycans, where each row represents a glycan and each column represents a lectin; *GF*_*i,k*_ indicates the expressed glycan feature *k* on glycan *i*; and *W*_*k,j*_ is lectin binding rules that tells the frequency of glycan feature *k* recognized by lectin *j*. In this study, we assume that glycan features are recognized by their occurrence counts. Binding rules were adapted from literature (48), where they provided *p*-values for significant epitopes bound by each lectin. We converted the *p* values to z-score, and used it as our binding rule matrix. The strength of the binding rules is reflected through the z-scores: high z-scores indicate strong binding affinities, as they correspond to low *p*-values, indicating significant and reliable binding interactions; lower z-scores on the other hand, correspond to higher *p*-values, suggesting less significant binding interactions (Supplementary Data 3).

(2) $${Lpg}_{n,j}={Gpg}_{n,i}*{Lpg}_{i,j}$$

*LPg*_*n,j*_ is the lectin binding profiles for given GPs, where each row represents a specific GP, and each column represents a lectin; *GPg*_*n,i*_ is the signal intensity (relative MS/HPLC/UPLC relative area under peak) of glycan *i* in the given GP *k*.

To calibrate the simulation, we performed regression fitting using the sklearn.linear_model.LinearRegression function. Regression analysis was used to calculate the estimated coefficients and intercepts for each lectin using the measured and simulated LPs from our IgG and Fetuin B samples. Finally, we applied this linear fitting to *LPg*_*n,j*_ to correct the LP simulation.

### Ambiguous reconstruction from lectin profile

To demonstrate the problem of mapping a single lectin binding profile to a specific GP, we repeatedly sample random GPs (74 glycans) from a symmetric Dirichlet prior, set entries < 0.01 to zero, and renormalize to sum to 1. For each sampled GP, we generate a simulated LP using our validated simulator; apply the standard rule-based post-processing from our pipeline, and renormalize. Taking the first simulated LP as the anchor, we continue sampling (with incremented random seeds for reproducibility) and retain simulations whose LPs have an absolute Pearson correlation > 0.95 with the anchor. Once 100 such profiles are collected (this requires the generation of 4722 different GPs), we plot a heatmap of their corresponding GPs to illustrate that distinct glycan compositions can yield nearly indistinguishable lectin readouts.

### Model tuning and training

The LeGenD ANN was trained using 309 experimentally measured N-GPs obtained from 10 recombinant glycoproteins produced in 30 geCHO cell lines, as described above. Each sample was represented by the relative abundance of 74 distinct N-glycans, while its input vector comprised eight lectin-binding intensities simulated from published specificity data. All preprocessing and model training were performed in Python 3.9 using TensorFlow 2.15.0.

We developed a neural network model using LP data as input, where lectins were represented as features in the rows. The model produced multiple continuous values corresponding to the relative abundance fraction of each glycan. Model training was constructed using the TensorFlow framework, with RMSprop as the optimizer and softmax activation for the output layer. We trained the model on 80% of our training dataset, using a batch size of 4 for 200 epochs, and explored all possible combinations within predefined hyperparameter ranges (Fig. S3 and Table S2) to identify the optimal set for the best model performance. To ensure robust evaluation and mitigate overfitting, we employed 3-fold cross-validation during hyperparameter tuning. The dataset was partitioned into three subsets, with the model trained on two subsets and validated on the remaining subset in a rotating manner. This procedure was repeated three times, ensuring each subset served as the validation set once. The average RMSE across the three folds was used to determine the optimal hyperparameter combination. This entire process was repeated 10 times to evaluate the average performance.

A threshold was established to determine meaningful improvements in the model's loss value. When the loss value decrease was less than 0.002, based on the statistical significance within the 95% confidence interval, the model was not considered to have a significant improvement. This threshold ensured that only statistically significant improvements were acknowledged. The model configuration with four hidden layers and 20 nodes yielded the best performance (loss value bolded in Table S2). The relationship between model performance and computational cost is visualized in Fig. S2. As shown, computation time consistently increases with the number of layers and nodes in the model. Notably, the steepness of the trend lines indicates that the 4-layer models strike a good balance between complexity and performance, making them particularly effective for the task at hand. This observation further supports our decision to select the model with 4 layers and 20 nodes.

Utilizing the identified optimal hyperparameters, we constructed 10 replicate neural network models to analyze IgG and Fetuin B samples. Each model was trained using the full training dataset. The architecture of our models consisting of 4 layers with 20 neurons each, and the rectified linear unit was employed as the activation functions. The learning rate was set at the default value of 0.001, and Mean Squared Error was used as the loss function. To ensure robustness and generalizability, we conducted 500 training epochs for each replicate model. For reproducibility, we initialized each model with a randomly generated seed, which also governed the data shuffling process.

### Confidence metric for glycan prediction

To aid interpretation of individual glycan predictions, we assigned each LeGenD prediction a model-intrinsic confidence tier. The ANN outputs a softmax-normalized probability vector across all glycans in the prediction space. Because low-abundance glycans can receive small residual probabilities, we used both the predicted probability and the relative separation between adjacent model outputs to estimate prediction confidence.

Predicted probabilities were converted to relative logit scores by taking the natural logarithm of each probability, with a small floor value of 10^-10^ to avoid undefined values. Although these recovered logits are defined only up to an additive constant, differences between logits are preserved and reflect the relative strength of model support for competing glycans. Glycans were ranked by descending relative logit score, and the logit gap for each glycan was calculated as the difference between that glycan and the next lower-ranked glycan.

Each prediction was then assigned to a high-, medium-, or low-confidence tier using fixed thresholds. High-confidence predictions were defined as glycans with strong predicted abundance and clear separation from the next lower-ranked prediction. Low-confidence predictions were defined as non-trivial glycan calls with poor logit separation, indicating that the model did not clearly distinguish that glycan from competing predictions. All remaining predictions were assigned as medium confidence. Specifically, high-confidence predictions required a logit gap of at least 0.75 and predicted abundance of at least 0.10, whereas low-confidence predictions were assigned when the logit gap was below 0.30 for glycans with predicted abundance of at least 0.02.

These thresholds were applied uniformly across all glycans and samples. The confidence labels were derived entirely from the model output and were not assigned by comparison to the experimentally observed GPs. Thus, the confidence annotation provides a prospective guide for interpreting LeGenD predictions, particularly for distinguishing dominant, well-supported glycans from lower-confidence predictions that should be interpreted cautiously.

### Model output collection and normalization

The outputs generated by each model were systematically collected and normalized using a cutoff threshold of 0.02. To identify the optimal threshold, we evaluated a range of cutoff thresholds (0, 0.01, 0.02, 0.03, 0.05, 0.1) against two key performance metrics: Root Mean Square Error (RMSE) and accuracy. RMSE measures the average magnitude of error between predicted and actual values, providing insight into overall prediction error, while the accuracy metric assesses the binary classification performance of the models. By converting predictions into binary 0/1 outputs, we calculated the percentage of correctly predicted presences or absences of the glycans, irrespective of quantity.

The model performance at different thresholds is detailed in Table S3. Our analysis determined that a threshold of 0.02 provided the best balance between RMSE and accuracy. Consequently, the threshold was selected for normalizing model outputs in subsequent analyses.

### SHapley additive explanations analysis

We performed SHAP analysis using the KernelExplainer functions from SHAP library (version 0.41.0) (47). SHAP provides local interpretation, so we randomly selected one sample from the rhA1AT and pdA1AT data for analysis. SHAP values of all the eight lectins were determined. As we trained and tested 10 replicate models, the SHAP values are averages derived from all 10 models. These SHAP values, either positive or negative, delineate the extent of their impact within the model. This relationship between the relative abundance of certain lectin-glycan interactions in a given LP and their correlation with a given *N*-glycan structure is effectively illustrated by the corresponding bipartite network diagrams (Fig. 5, *A* and *B*).

### Statistical analyses

All statistical calculations were implemented in Python (v3.9; https://www.python.org). For comparing LeGenD predictive performance with UPLC-derived GPs, we used RMSE. To determine the variability of the predictions across the replicate models, we used standard error. Pearson correlation analysis was employed to assess the similarities between the simulated LPs. Pearson correlation coefficients were reported to quantify the magnitude and the direction of their linear correlations. KDE plots (Supplementary Data 4) were generated using the Seaborn (v0.13.1; https://seaborn.pydata.org/) library. The glycan structures were drawn using GlycoGlyph (112) (https://glycotoolkit.com/Tools/GlycoGlyph/)

## Data availability

The authors declare that all other data supporting the findings of this study are available within the paper and its supplementary information files.

## Code availability

The computing environment was standardized across all experiments, running on Apple M2 chip, with our models implemented using TensorFlow version 2.15.0. All code can be obtained here: https://github.com/lewiscelllabs/LeGenD/tree/main/LeGenD-main.

## Supporting information

This article contains supporting information (48, 65, 87, 113, 114, 115, 116, 117, 118, 119, 120, 121, 122, 123, 124, 125, 126, 127).

## Conflict of interest

AWTC and NEL are inventors on a patent associated with this study. The remaining authors declare no competing interests.
