# Supplementary material for: LeGenD: High-throughput N-glycan profiling using explainable AI and lectin profiling
Source: J Biol Chem. 2026 Jun 4;302(7):113234. doi: 10.1016/j.jbc.2026.113234 (PMC13330679; doi:10.1016/j.jbc.2026.113234)
Supplement: Supporting Figures and Tables [file mmc1.docx]

Supplementary Materials for

**LeGenD: high-throughput *N*-glycan screening using explainable AI and lectin profiling**

Kiki H. Li *et al*

*Corresponding author. Email: [auchiang@augusta.edu](mailto:auchiang@augusta.edu), [nlewisres@ucsd.edu](mailto:nlewisres@ucsd.edu)

**This PDF file includes:**

Figs. S1 to S3

Tables S1 to S2

Data S1 to S5

Fig. S1.

**A B**


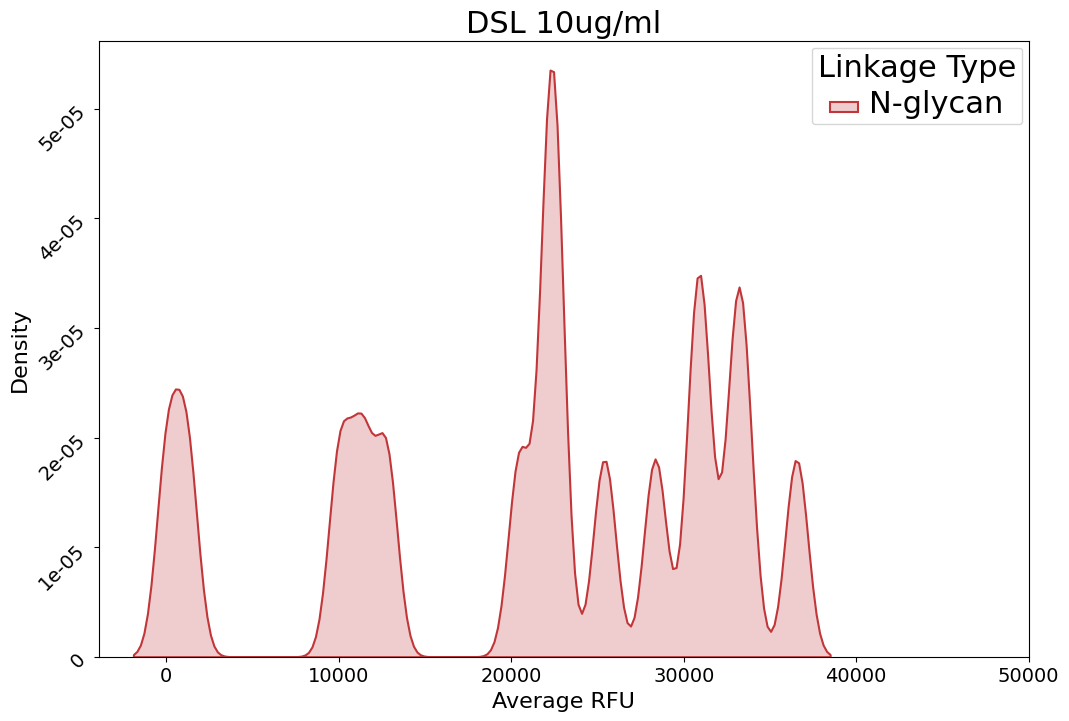

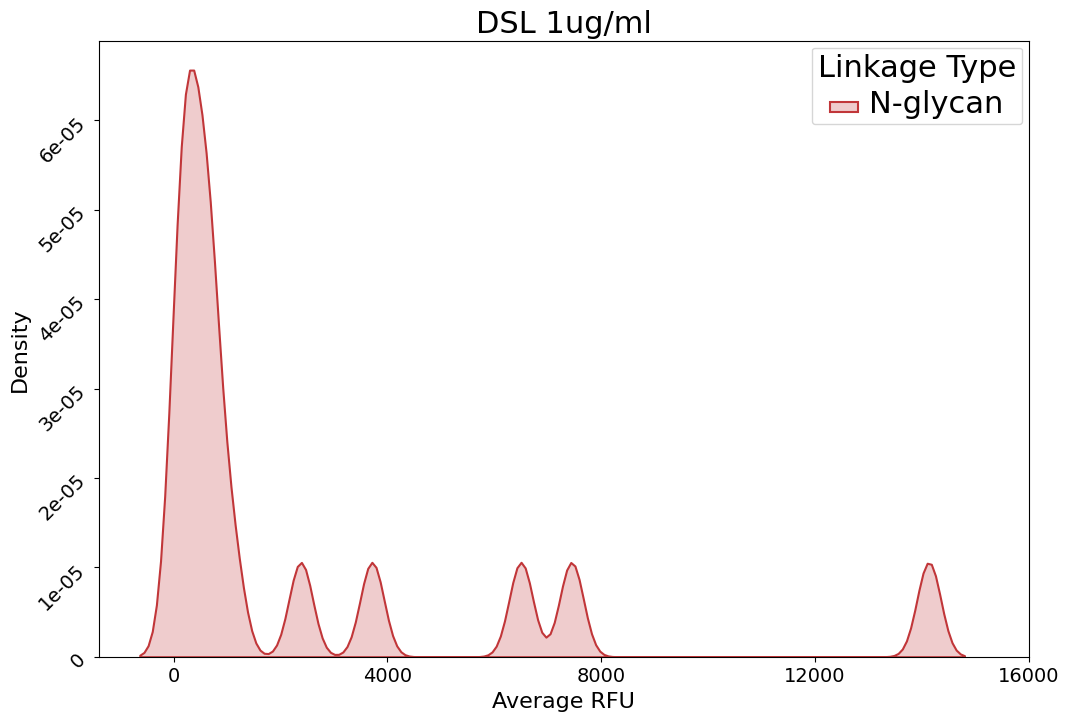


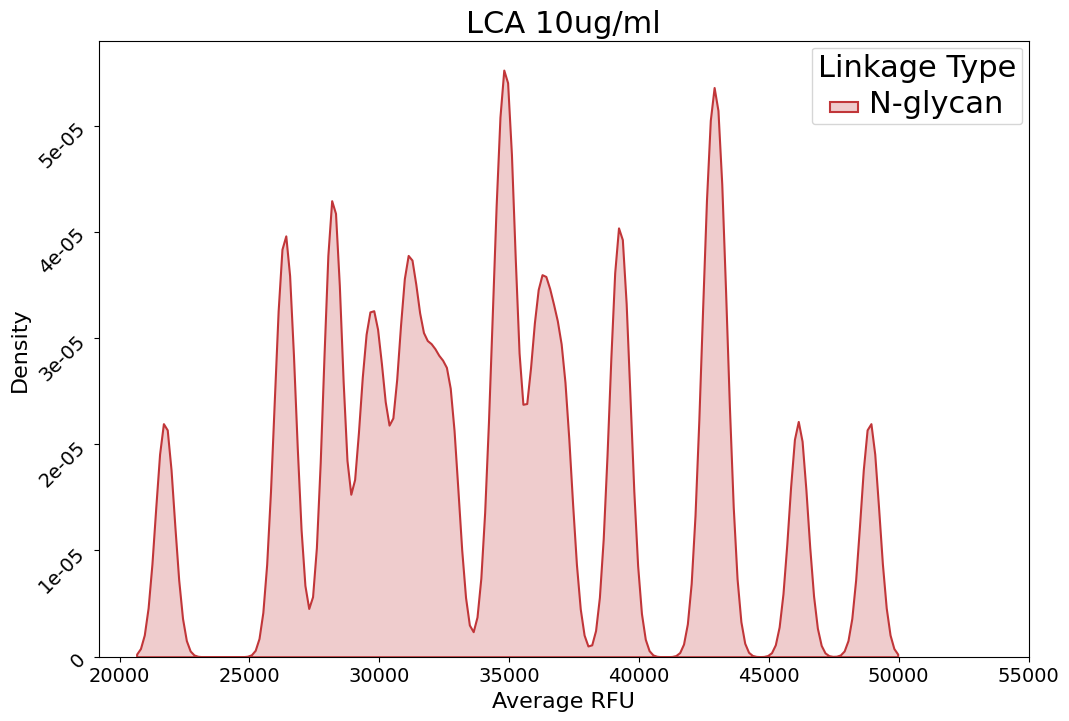

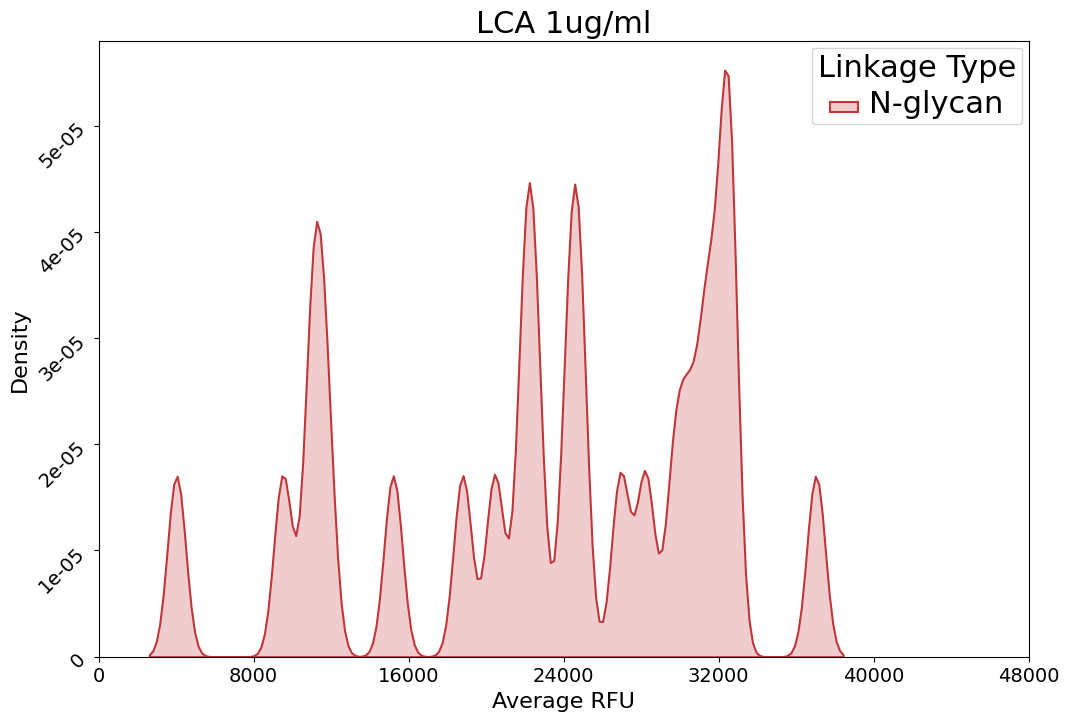


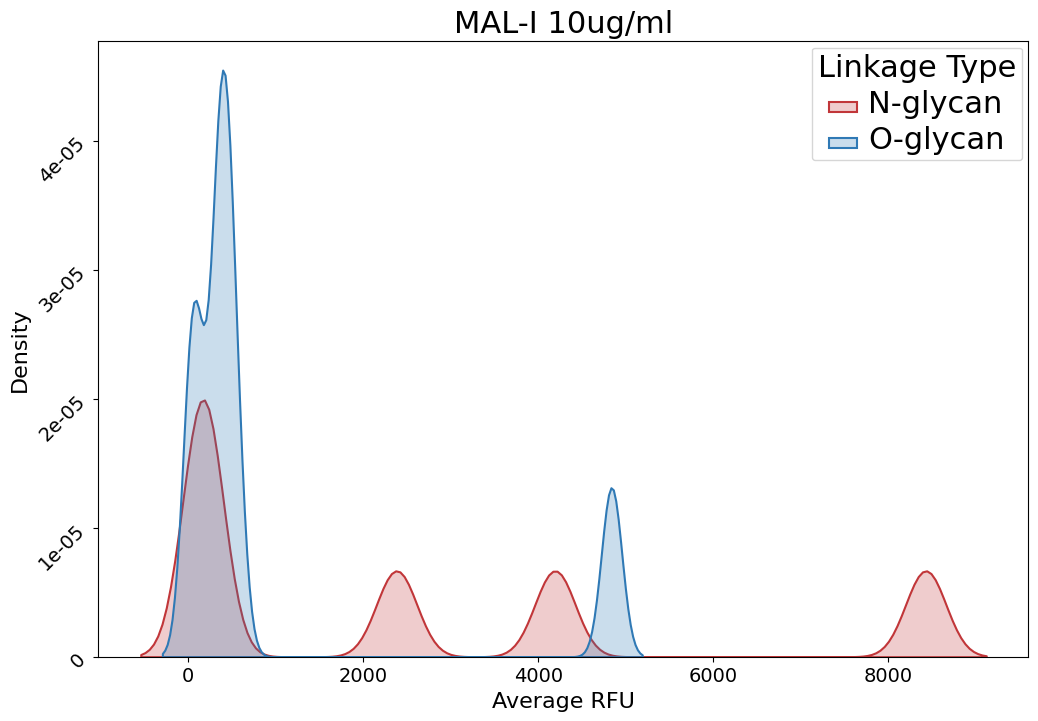

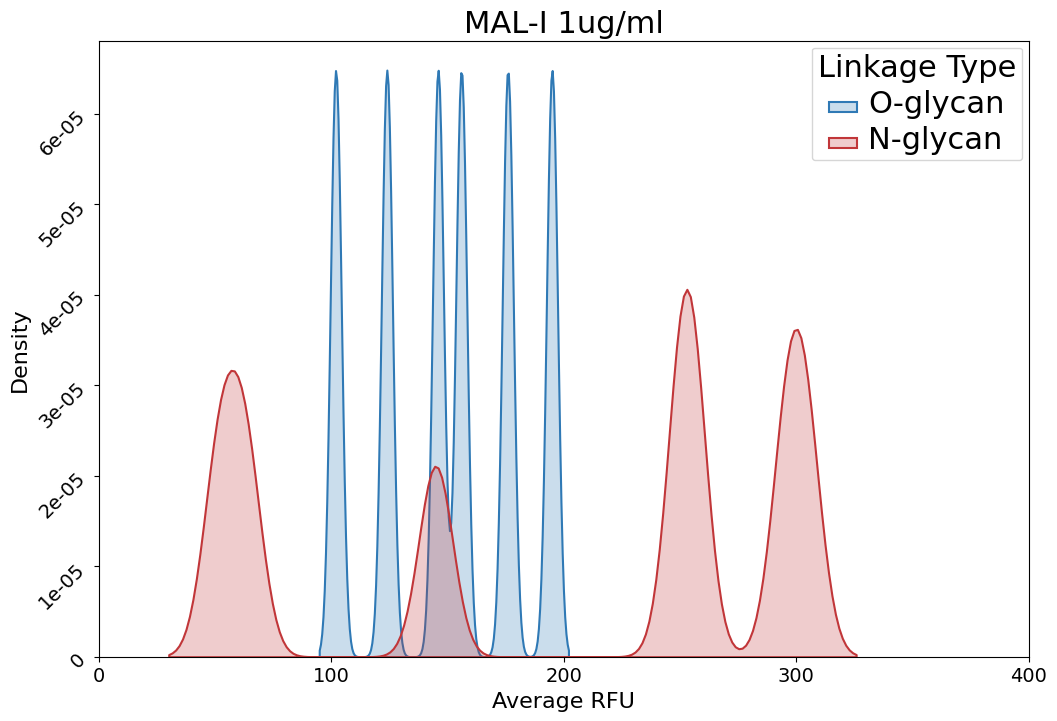


**
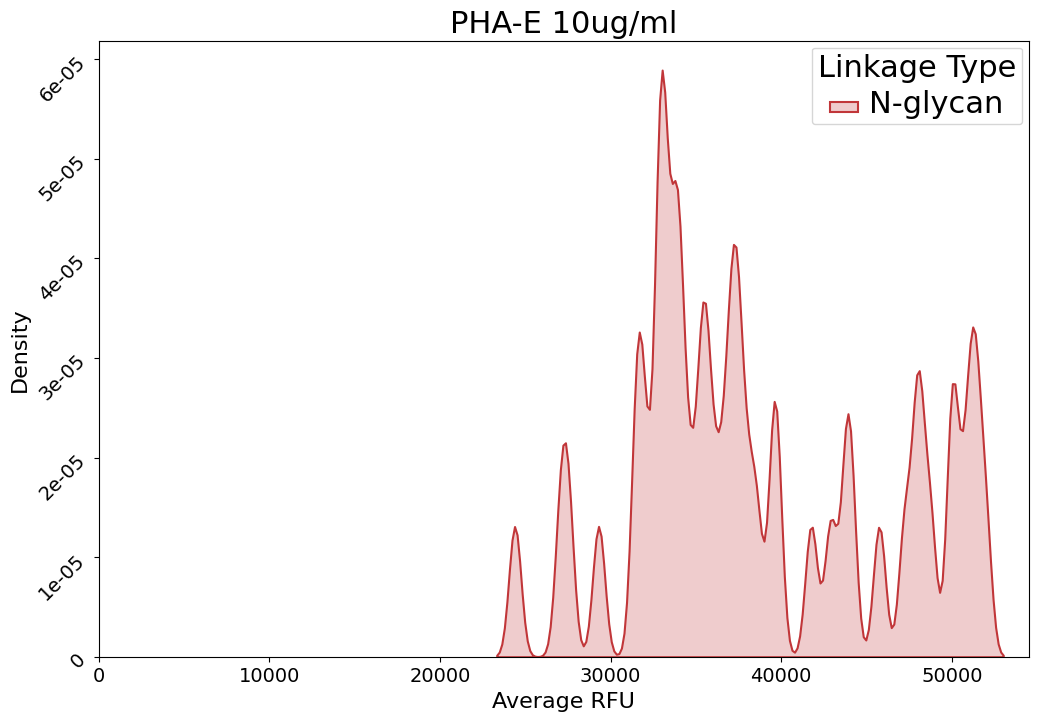

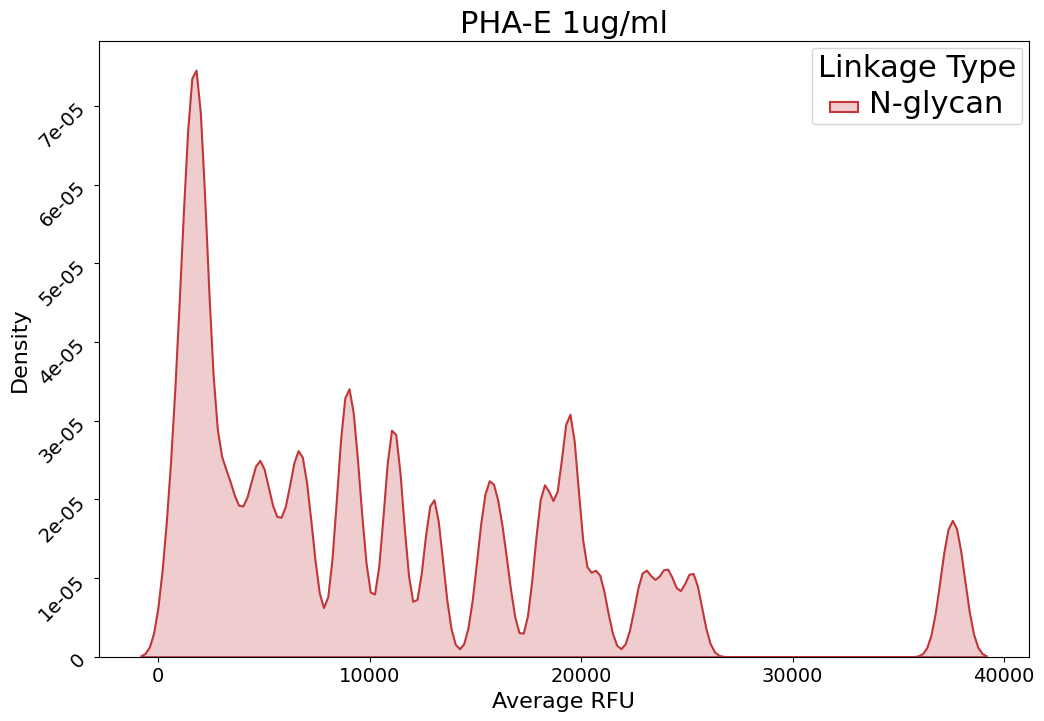
**

**
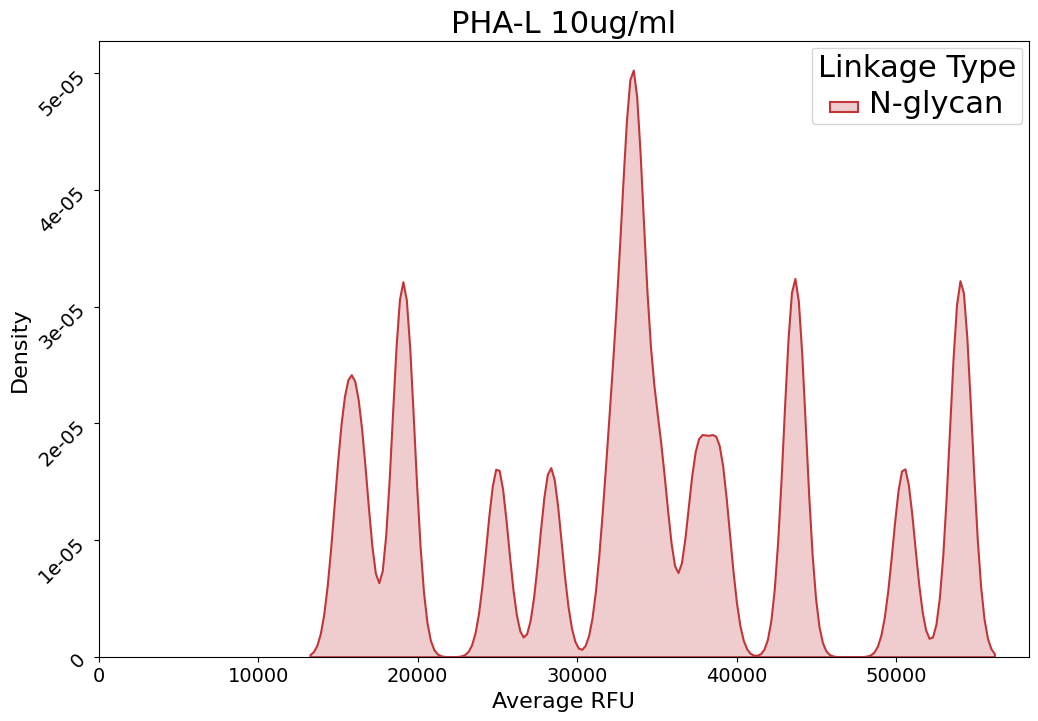

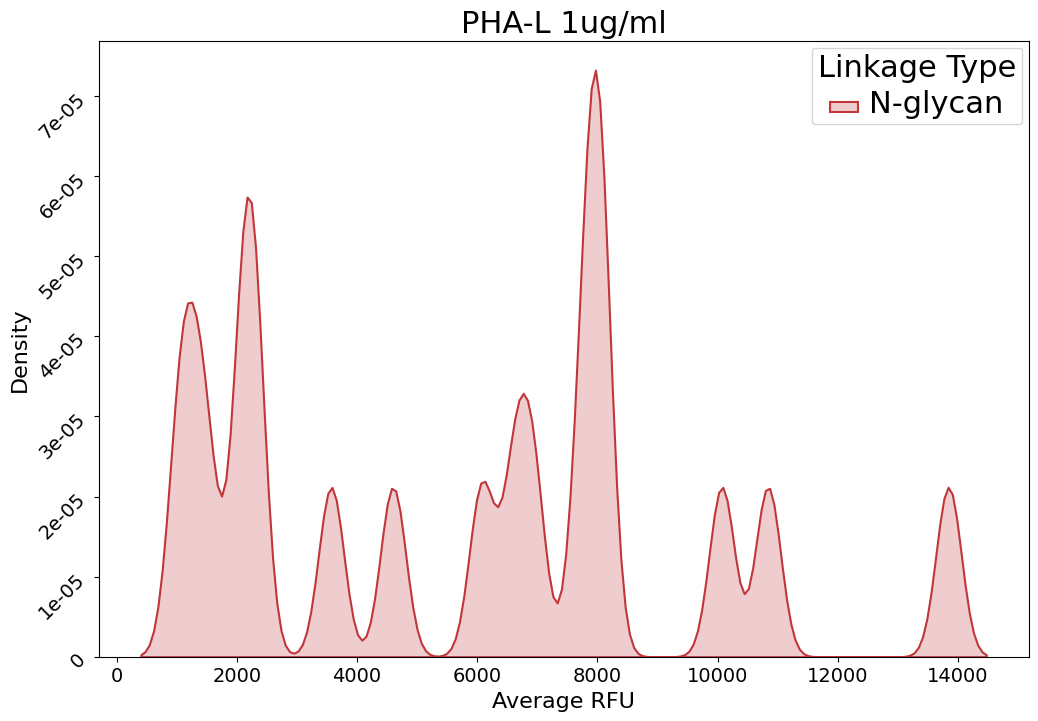
**

**
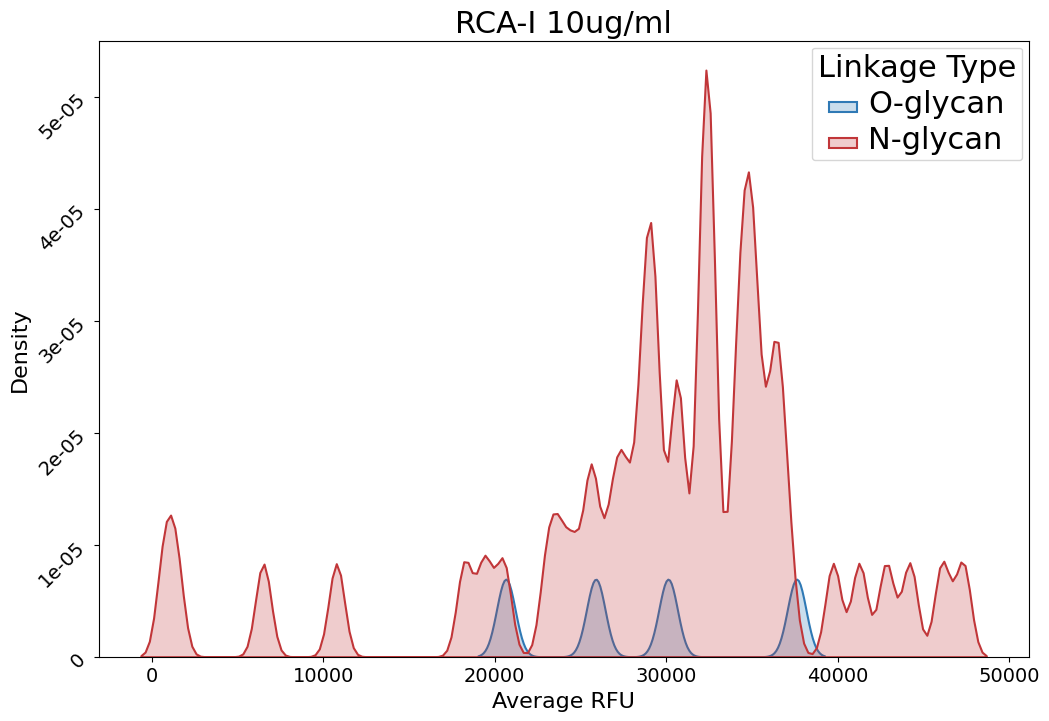

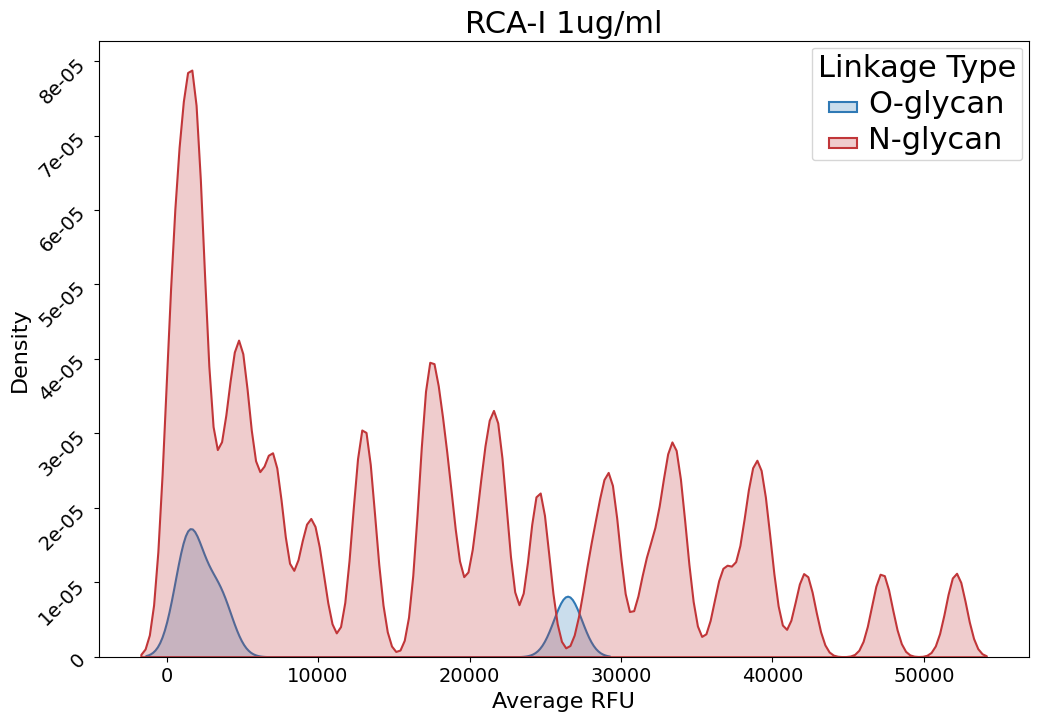
**

**
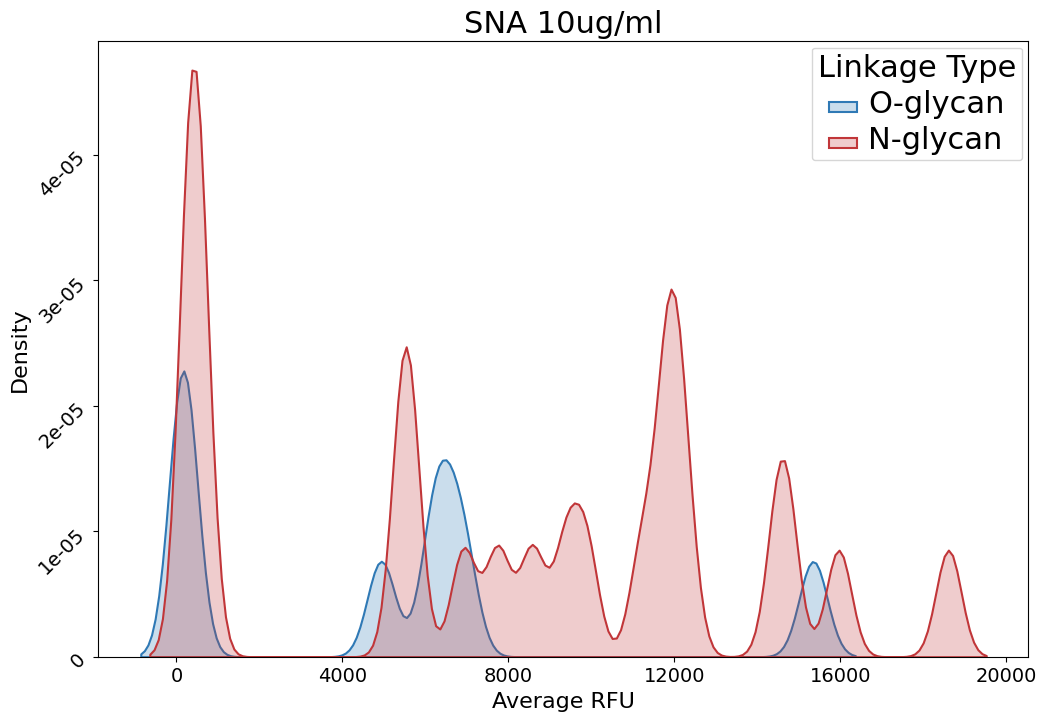

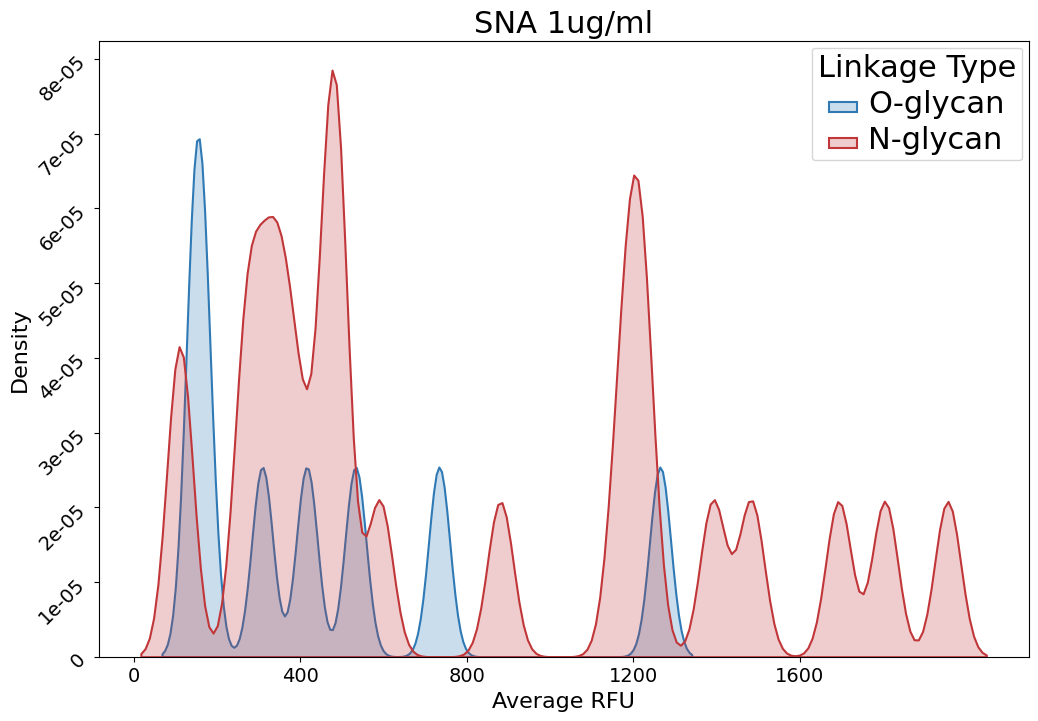
**

**
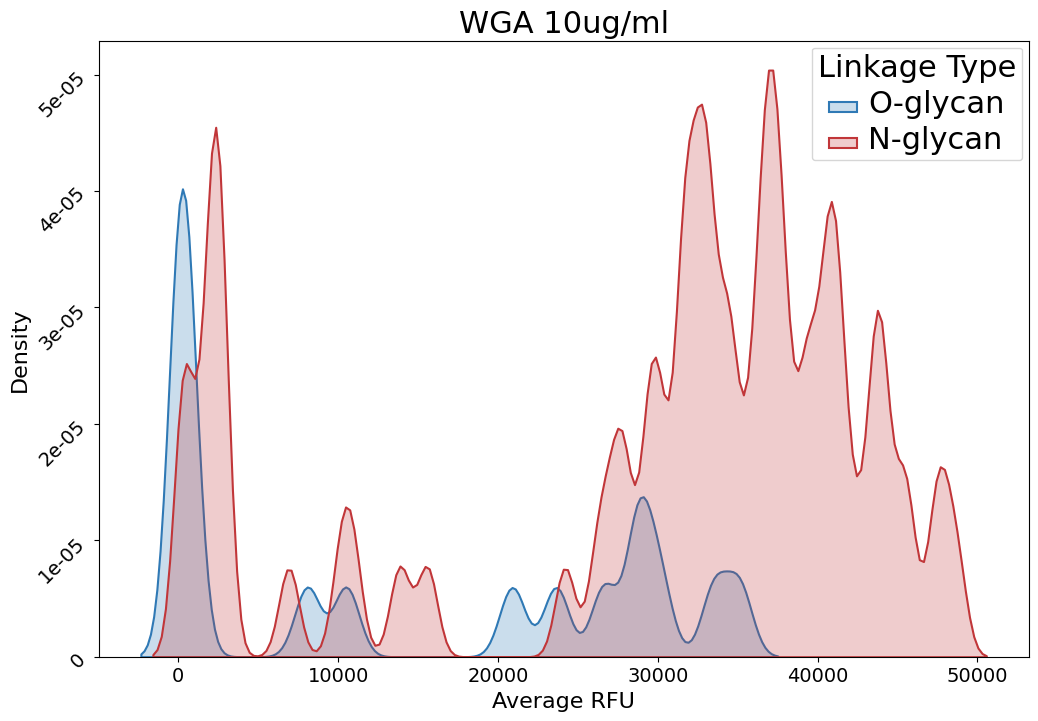

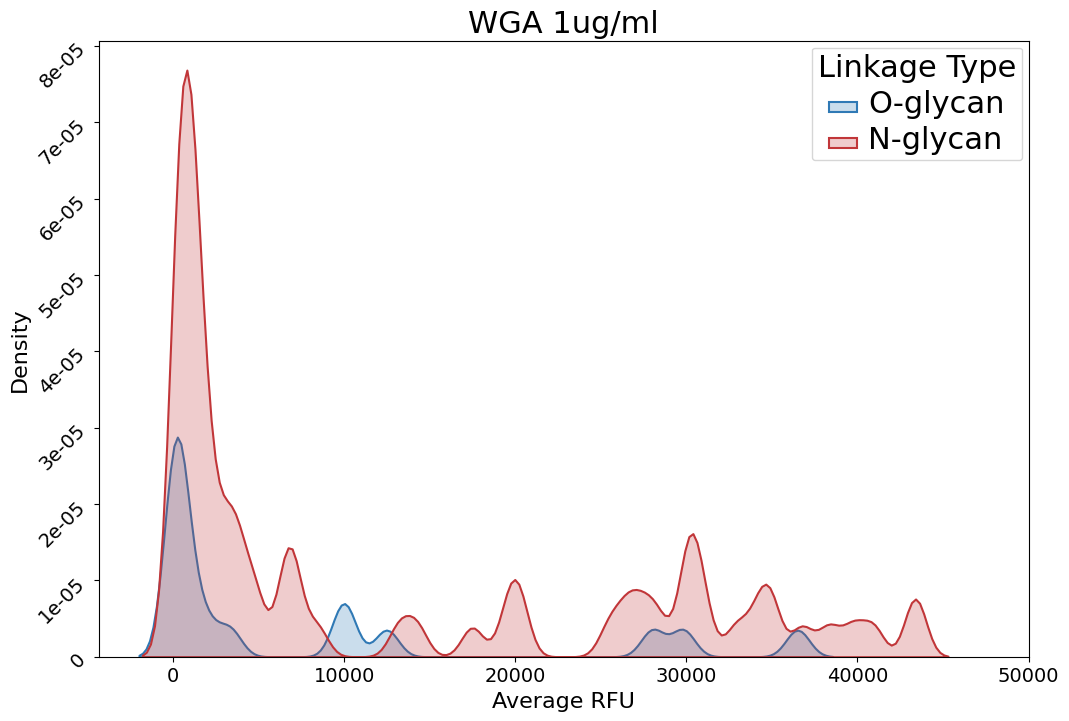
**

**Kernel density estimate (KDE) plots illustrating the specificity of eight (8) lectins towards N-glycan and O-glycan epitopes as employed in LeGenD.** CFG microarray data from biotinylated lectins at concentrations of **A** 10µg/ml and **B** 1µg/ml were analyzed to determine the distribution of N-glycan and O-glycan epitopes targeted by the lectins used in LeGenD (rows). The Average Relative Fluorescence Units (RFU) of lectin signals were plotted, with peaks indicating binding signals for either *N*-glycan (red) or *O*-glycan (blue) epitopes. Higher peak density corresponds to a greater concentration of epitopes at a given Average RFU level.

Fig. S2.

**
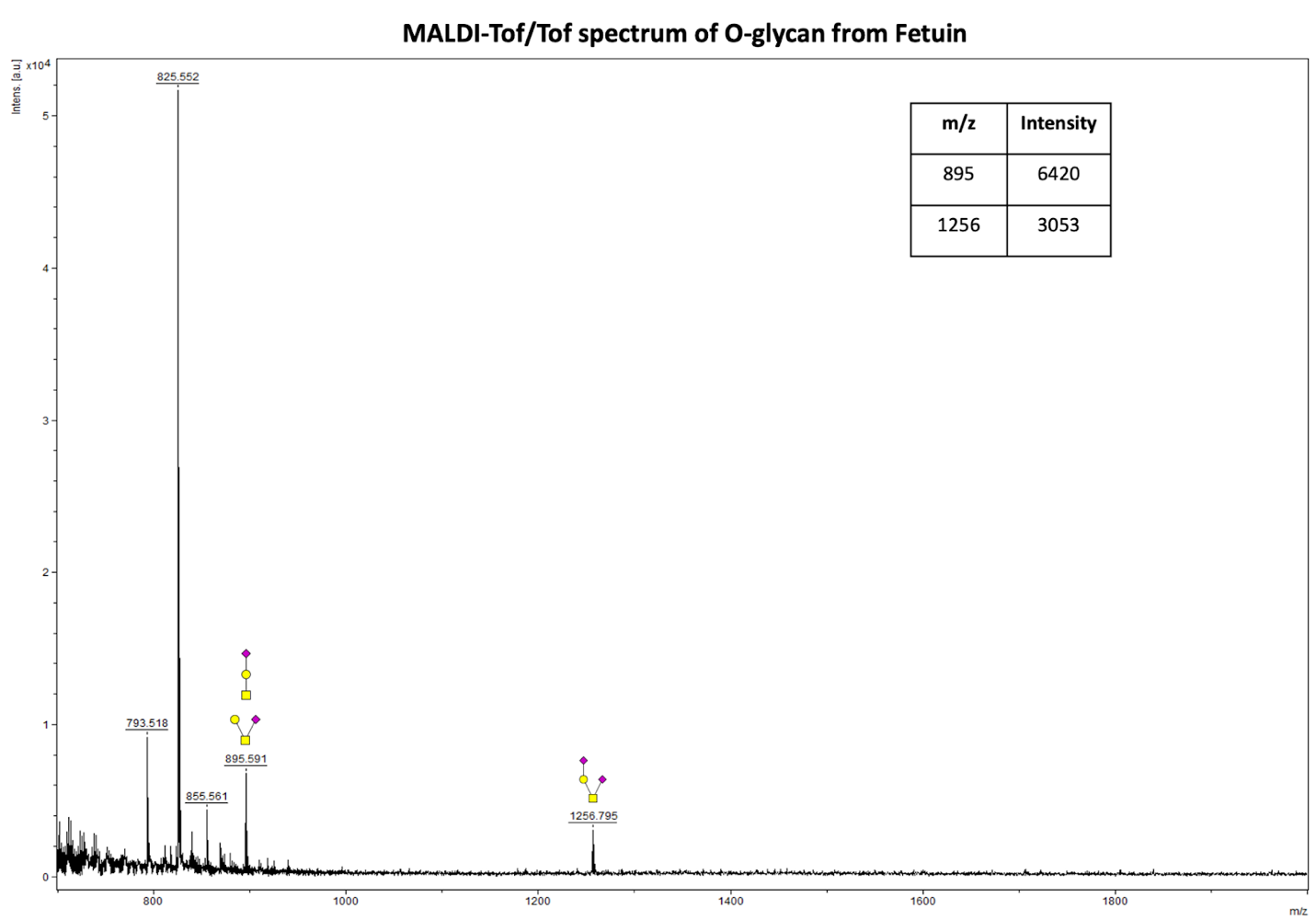
**

**MALDI-TOF O-glycan profile of Fetuin B**

Representative MALDI-TOF mass spectrum of O-linked glycans released from bovine fetuin B. Detected species correspond predominantly to mono-sialylated and di-sialylated Core 1 O-glycans. Peak assignments are based on observed m/z values.

Fig. S3.

**
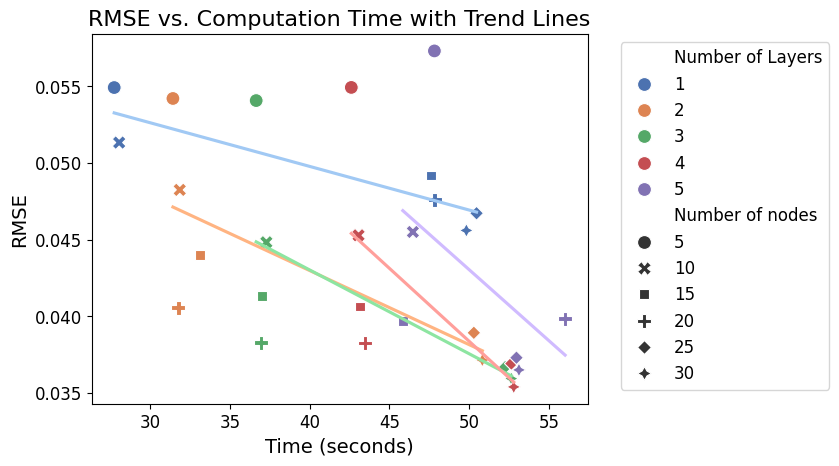
**

**Scatter plot illustrating how computation time increases as the model gets more layers/nodes.** The time reported is for training each model with 80% of the samples over 200 epochs. The different markers represent models with varying numbers of nodes, while the colors correspond to different numbers of layers. Trend lines are generated using linear regression to visualize the relationship between computation time and the number of nodes. The steepness suggests that the 4-layer models balance complexity and performance well.

**Table. S1.**

| **Lectins** | **Full Name** | **Preferred *N*-Glycan Epitopes** | **SNFG Icon** |
| --- | --- | --- | --- |
| DSL | Datura Stramonium Lectin | Chitin[^1,2^](https://paperpile.com/c/KtY8bw/H1RHp+aPGZe) | 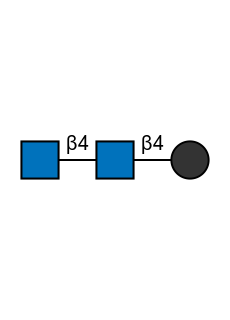 *n |
|  |  | Type II poly LacNAc[^3,4^](https://paperpile.com/c/KtY8bw/LqCXW+7yd1D) | 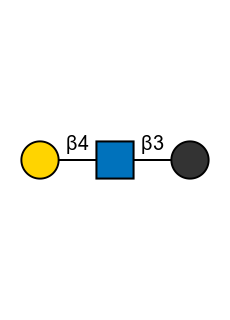*n |
| LCA | Lens Culinaris Agglutinin | Core fucose[^5^](https://paperpile.com/c/KtY8bw/0T6y3) | 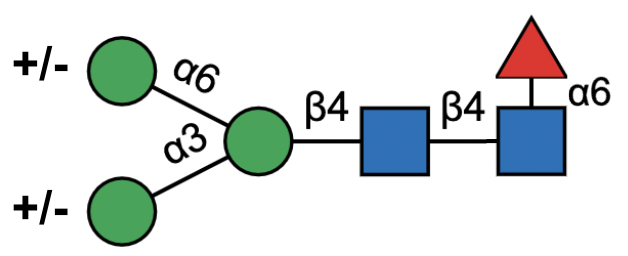 |
|  |  | Terminal Man ɑ1,2[^5^](https://paperpile.com/c/KtY8bw/0T6y3) | 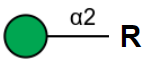 |
| MAL-I | Maackia Amurensis-I | ⍺2,3 sialylated LacNAc[^6^](https://paperpile.com/c/KtY8bw/lCUDt) | 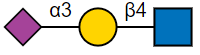 |
|  |  | Terminal 3-O sulfated Gal on LacNAc[^6^](https://paperpile.com/c/KtY8bw/lCUDt) | 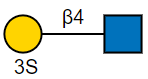 |
| PHA-E | Phaseolus Vulgaris Erythroagglutinin | Bisecting GlcNAc[^7^](https://paperpile.com/c/KtY8bw/9hbrm) | 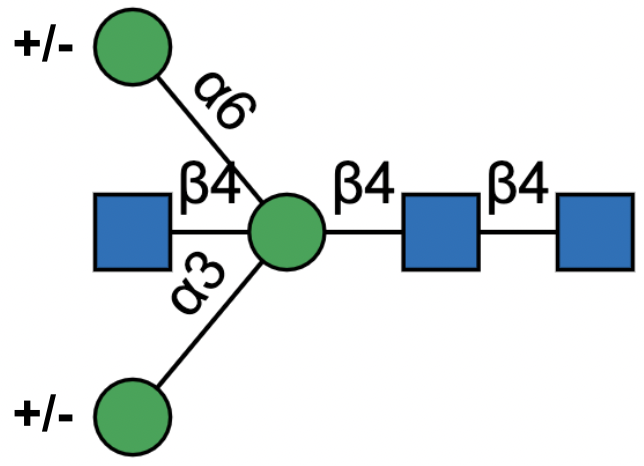 |
|  |  | Bi/Tri-antennary[^8–10^](https://paperpile.com/c/KtY8bw/Xvsgk+KXtI6+ZWJ3v) | 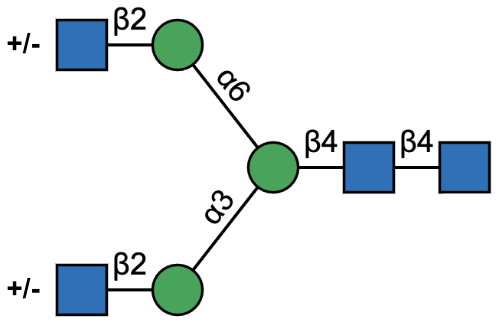  or  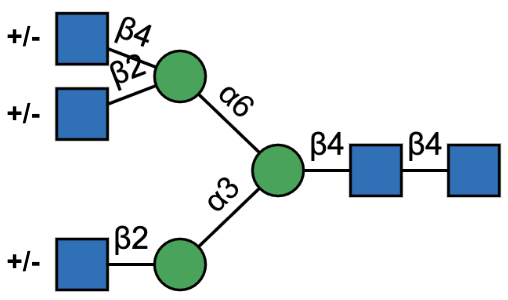 |
|  |  | Galactosylated[^8–10^](https://paperpile.com/c/KtY8bw/Xvsgk+KXtI6+ZWJ3v) | 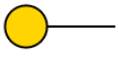 |
| PHA-L | Phaseolus Vulgaris Leucoagglutinin | β1,6-branched[^8,9^](https://paperpile.com/c/KtY8bw/KXtI6+Xvsgk)^-^[^7,11^](https://paperpile.com/c/KtY8bw/9hbrm+PhsHL) | 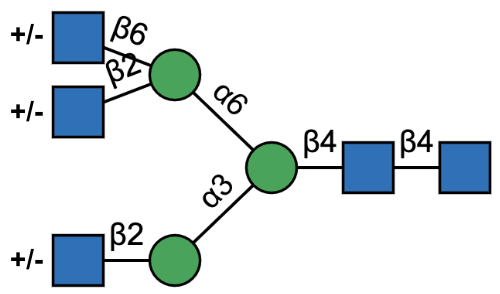 |
|  |  | Tri/Tetra-antennary[^12^](https://paperpile.com/c/KtY8bw/Abrgj)^,^[^10^](https://paperpile.com/c/KtY8bw/ZWJ3v) | 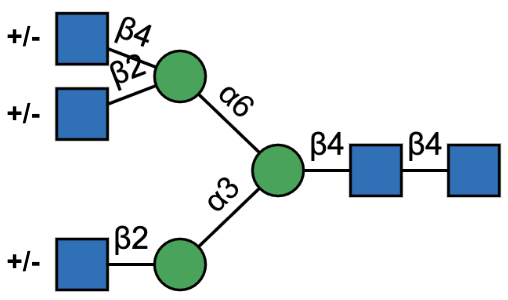 or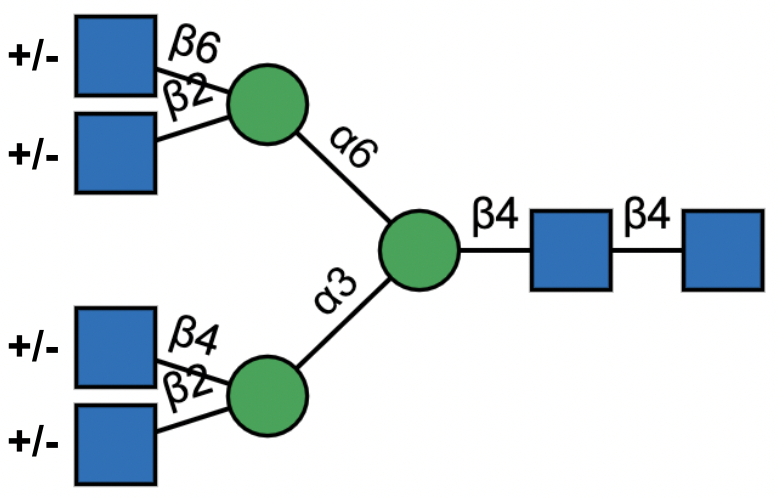 |
|  |  | Galactosylated[^10^](https://paperpile.com/c/KtY8bw/ZWJ3v) | 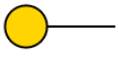 |
| RCA-I | Ricinus Communis Agglutinin I | Terminal type 2 LacNAc[^13^](https://paperpile.com/c/KtY8bw/SyAEd) | 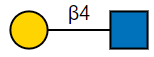 |
|  |  | Galβ1,4[^13,14^](https://paperpile.com/c/KtY8bw/SyAEd+UzVK1) | 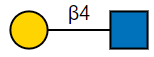 |
| SNA | Sambucus Nigra Agglutinin | α2,6-sialic acid[^15^](https://paperpile.com/c/KtY8bw/qWuIt) (Neu5Ac α2,6[^16^](https://paperpile.com/c/KtY8bw/hI5P9)) | 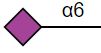 |
| WGA | Wheat Germ Agglutinin | Terminal GlcNAc[^17^](https://paperpile.com/c/KtY8bw/1Dzg9) | 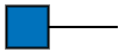 |
|  |  | Sialic acid[^18^](https://paperpile.com/c/KtY8bw/MbMpQ) | 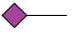 |
|  |  | Biantennary[^17^](https://paperpile.com/c/KtY8bw/1Dzg9) | 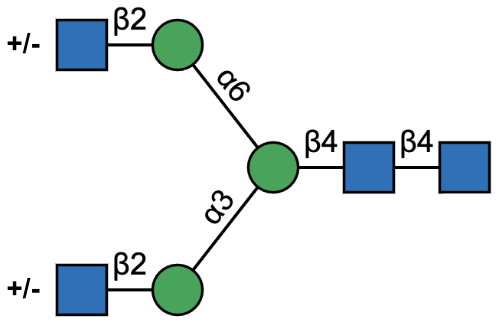 |
|  |  | 2,2,6-form triantennary[^17^](https://paperpile.com/c/KtY8bw/1Dzg9) | 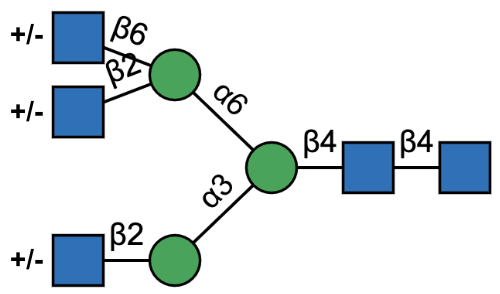 |

**Selected lectins for *N*-glycan lectin profiling**

**Table. S2.**

|  | | Neurons per layer | | | | |
| --- | --- | --- | --- | --- | --- | --- |
|  |  | 5 | 10 | 15 | 20 | 25 |
| Number of Layers | 1 | 0.055 | 0.051 | 0.049 | 0.048 | 0.047 |
|  | 2 | 0.054 | 0.048 | 0.044 | 0.041 | 0.039 |
|  | 3 | 0.054 | 0.045 | 0.041 | 0.038 | 0.037 |
|  | 4 | 0.055 | 0.045 | 0.041 | **0.038**^^[[1]](#footnote-1)^^ | 0.037 |
|  | 5 | 0.057 | 0.046 | 0.040 | 0.040 | 0.037 |

**RMSE values of the model when tuning the hyperparameters.**

Supporting information (separate file)

N-glycan and O-glycan glycoprofiling

Supporting Data 1. (separate file)

Training glycoprofile data

**Supporting Data 2. (separate file)**

Experimental ELISA data

**Supporting Data 3. (separate file)**

KDE analysis

**Supporting Data 4. (separate file)**

Lectin binding rules z-scores

1. Best performing model architecture [↑](#footnote-ref-1)
