# Supplementary material for: LeGenD: High-throughput N-glycan profiling using explainable AI and lectin profiling
Source: J Biol Chem. 2026 Jun 4;302(7):113234. doi: 10.1016/j.jbc.2026.113234 (PMC13330679; doi:10.1016/j.jbc.2026.113234)
Supplement: Supporting Information [file mmc2.pdf]

Chromatogram and Results

Injection Details

Injection Name:	**HuIgG-20ug**	*Run Time (min):*	**70.00**
Vial Number:	**1:A2**	*Injection Volume:*	**10.00**
Injection Type:	**Calibration Standard**	*Channel:*	**ChanA_Emission**
Calibration Level:		*Wavelength:*	**n.a.**
Instrument Method:	**Procainamide-N-Glycan-70min-PMT5-091421**	*Bandwidth:*	**n.a.**
Processing Method:	**Proc-HuIgG-NGlycan-092223**	*Dilution Factor:*	**1.0000**
Injection Date/Time:	**22/Sep/23 15:25**	*Sample Weight:*	**1.0000**

Chromatogram

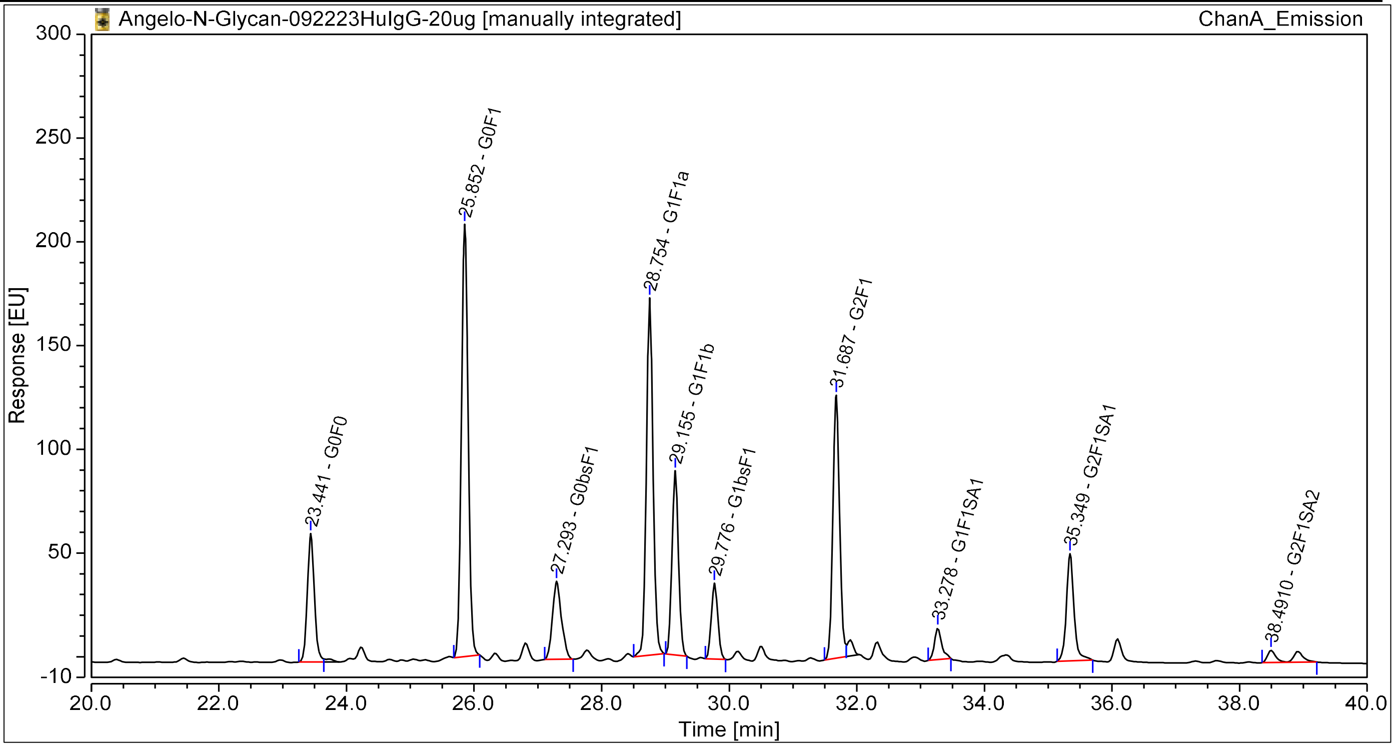

Integration Results

No.	Peak Name	Retention Time min	Area EU*min	Height EU	Relative Area %	Relative Height %	pmol
1	G0F0	23.440	7.899	61.862	7.79	7.69	n.a.
2	G0F1	25.853	25.159	208.323	24.81	25.88	n.a.
3	G0bsF1	27.293	6.182	37.617	6.10	4.67	n.a.
4	G1F1a	28.753	20.952	172.162	20.67	21.39	n.a.
5	G1F1b	29.152	10.571	88.761	10.43	11.03	n.a.
6	G1bsF1	29.768	4.317	36.555	4.26	4.54	n.a.
7	G2F1	31.677	15.398	126.772	15.19	15.75	n.a.
8	G1F1SA1	33.268	2.045	15.352	2.02	1.91	n.a.
9	G2F1SA1	35.342	7.098	51.532	7.00	6.40	n.a.
10	G2F1SA2	38.492	1.764	5.895	1.74	0.73	n.a.
**Total:**			**101.386**	**804.831**	**100.00**	**100.00**	**0.00**

Chromeleon (c) Dionex
Version 7.2.10.23925

Chromatogram and Results

Injection Details

Injection Name:	**Fetuin 40ug**	*Run Time (min):*	**70.00**
Vial Number:	**1:A2**	*Injection Volume:*	**10.00**
Injection Type:	**Unknown**	*Channel:*	**ChanA_Emission**
Calibration Level:		*Wavelength:*	**n.a.**
Instrument Method:	**Procainamide-N-Glycan-70min-PMT5-091421**	*Bandwidth:*	**n.a.**
Processing Method:	**Proc-NGlycan-111721**	*Dilution Factor:*	**1.0000**
Injection Date/Time:	**20/Oct/22 15:22**	*Sample Weight:*	**1.0000**

Chromatogram

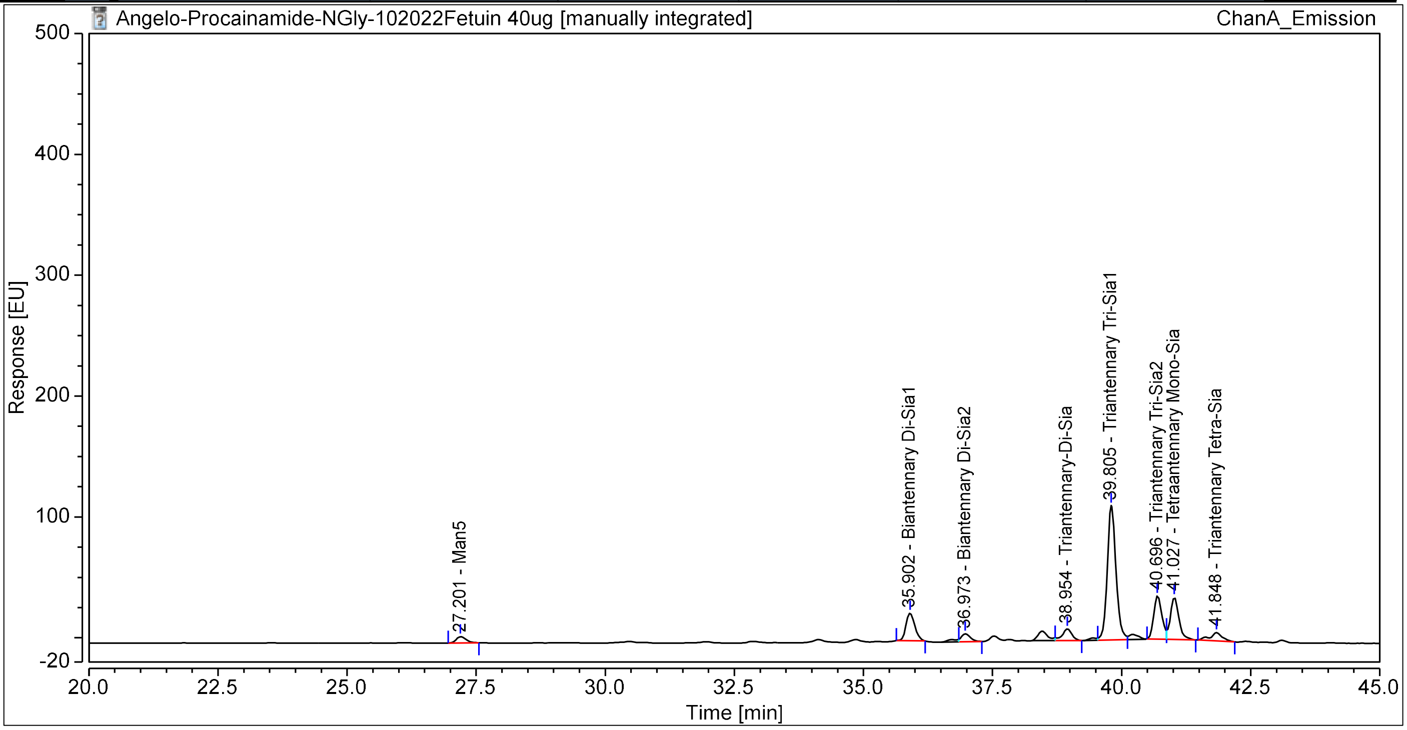

Integration Results

No.	Peak Name	Retention Time min	Area EU*min	Height EU	Relative Area %	Relative Height %	pmol
1	Man5	27.195	1.131	5.119	2.46	2.20	n.a.
2	Biantennary Di-Sia1	35.902	4.539	22.917	9.88	9.84	n.a.
3	Biantennary Di-Sia2	36.972	1.322	6.590	2.88	2.83	n.a.
4	Triantennary-Di-Sia	38.947	2.101	9.662	4.58	4.15	n.a.
5	Triantennary Tri-Sia1	39.800	21.681	111.096	47.21	47.71	n.a.
6	Triantennary Tri-Sia2	40.693	6.611	35.714	14.40	15.34	n.a.
7	Tetraantennary Mono-Sia	41.018	6.652	34.834	14.48	14.96	n.a.
8	Triantennary Tetra-Sia	41.837	1.887	6.930	4.11	2.98	n.a.
**Total:**			**45.924**	**232.862**	**100.00**	**100.00**	**0.00**

Chromeleon (c) Dionex
Version 7.2.10.23925

MALDI-Tof/Tof spectrum of O-glycan from Fetuin

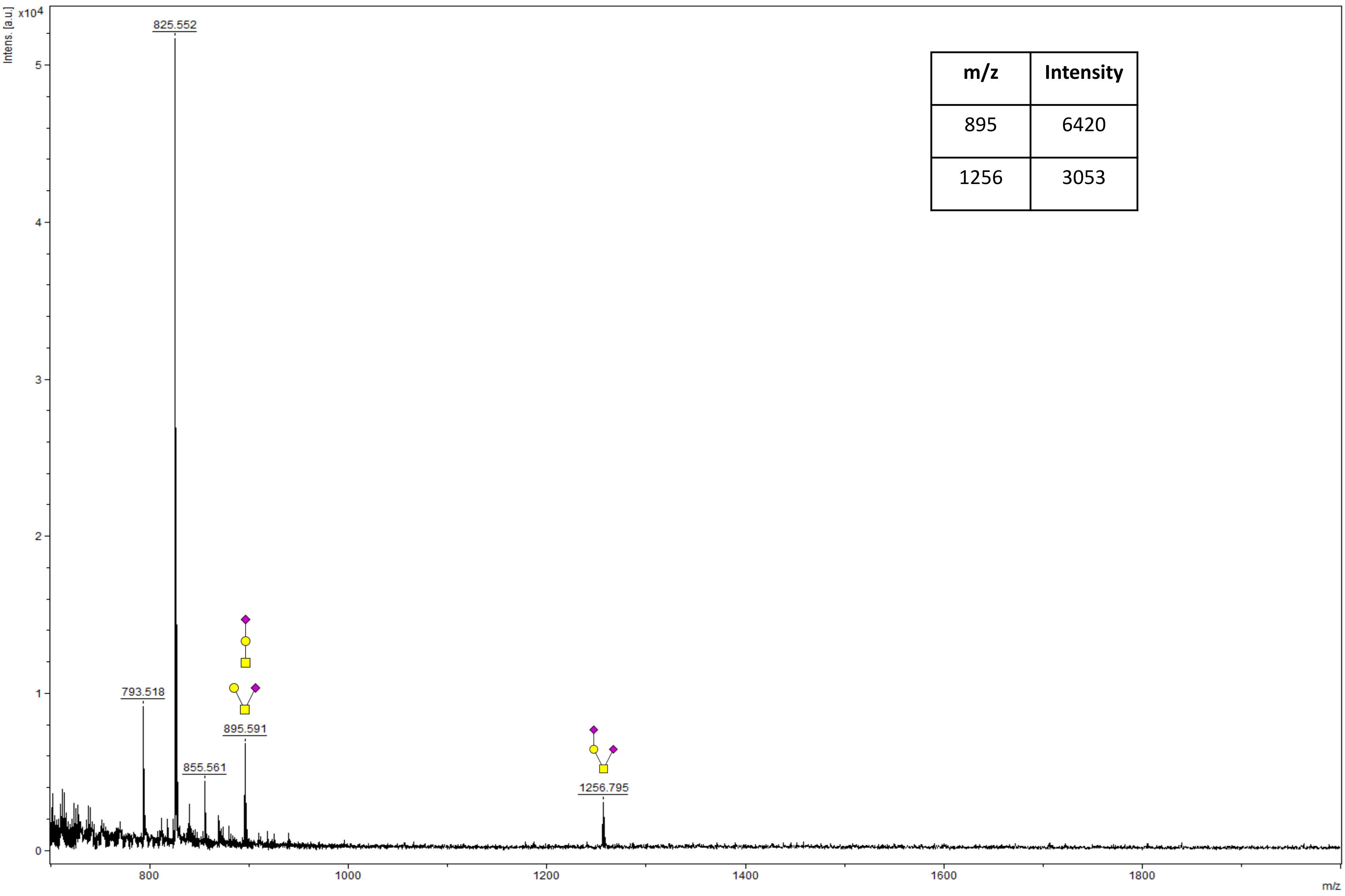

m/z	Intensity
895	6420
1256	3053
